# Machine learning framework for investigating nano- and micro-scale particle diffusion in colonic mucus

**DOI:** 10.1186/s12951-025-03659-6

**Published:** 2025-08-22

**Authors:** Marco Tjakra, Kristína Lidayová, Christophe Avenel, Christel A.S. Bergström, Shakhawath Hossain

**Affiliations:** 1 Department of Pharmacy, Uppsala Biomedical Center, Uppsala University, Uppsala, 751 23 Sweden; 2 The Swedish Drug Delivery Center, Department of Pharmacy, Uppsala University, Box 580, Uppsala, SE-751 6 23 Sweden; 3 Department of Information Technology, Uppsala University, Uppsala, Sweden; 4 BioImage Informatics Facility, Science for Life Laboratory, SciLifeLab, Sweden

**Keywords:** Mucus, Machine learning, Diffusion, Nanoparticles, Rheology

## Abstract

Biosimilar artificial mucus models that mimic native mucus facilitate efficient, lab-based drug diffusion studies, addressing the costly and challenging preclinical phase of drug development, especially for nano- and micro-scale particle-based colonic drug delivery. This study presents a machine-learning-driven framework that integrates microrheological features into diffusional fingerprinting to characterize nano- and micro-scale particle diffusion patterns in mucus and assess the effect of mucus microrheology on such movements. We investigated the diffusion of fluorescent-labeled polystyrene particles in native pig mucus and two artificial mucus models. Particles (100, 200, and 1000 nm in diameter) with carboxylate- or amine-modified surfaces were tracked during passive diffusion. From each particle trajectory, 20 features —including microrheology-based parameters— were extracted. Based on these features, seven supervised machine learning models were applied to classify or identify similarities among mucus hydrogels. Of these, gradient boosting achieved the highest accuracy. SHapley Additive exPlanations analysis identified creep compliance as the most influential feature in distinguishing the mucus models. In native mucus, smaller negatively charged nanoparticles exhibited the highest mobility, with fewer particles being in the immobile and subdiffusive states. Microrheology data further indicated that larger particles experienced greater restriction owing to the elastic properties of native mucus. In contrast, smaller particles interacted more with the viscous liquid phase. A comprehensive feature-wide analysis revealed that hydroxyethyl cellulose (HEC)-based artificial mucus more closely resembled native pig mucus than the polyacrylic acid-based model. In conclusion, the machine-learning-driven fingerprinting approach, incorporating microrheological features, successfully differentiated the microstructural characteristics and rheological properties of the three mucus models. It also supported the selection of HEC-based artificial mucus as a viable substitute for native colonic mucus.

**Graphical abstract:**

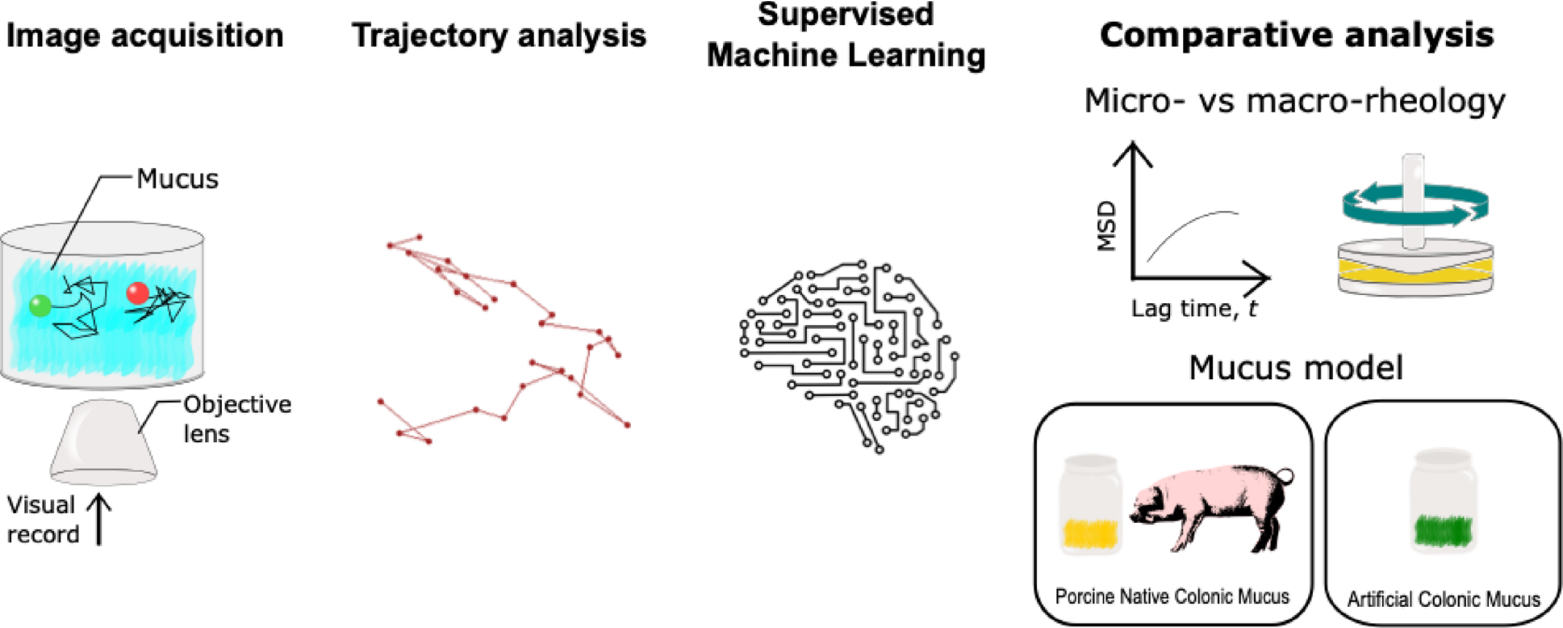

**Supplementary Information:**

The online version contains supplementary material available at 10.1186/s12951-025-03659-6.

## Introduction

Mucus is a hydrogel-like structure that lines the gastrointestinal tract, with mucin— secreted by epithelial cells—as its primary structural protein. The main functions of mucus are lubrication and protection [1]. Composed of glycosylated mucin, lipids, enzymes, metabolites, and nucleic acids, mucus forms a complex and highly heterogeneous structure [2]. This structural complexity imparts steric hindrance and enables size-selective filtering of particles, making mucus a critical barrier in drug delivery. In the colonic region, the mucus layer is considerably thicker than in other parts of the gastrointestinal tract, serving both as a protection and habitat for the dense gut microbiota [3]. However, while important for host defense, this dense and complex matrix also poses a challenge for drug delivery, as it can hinder drug diffusion and limit access to the underlying epithelial cells.

Drug transport through mucus—a biological hydrogel—occurs primarily via passive diffusion. This process depends on multiple parameters, including particle size, temperature, solvent viscosity, and Boltzmann’s constant, as described by the Stokes–Einstein equation. To enhance drug delivery to the colonic region, nano- and micro-scale particles have emerged as a promising strategy, with some nanoparticle-based drug formulations already approved by the United States Food and Drug Administration, including doxorubicin, DaunoXome^®^, and Thermodox^®^ [4]. Given the increasing use of nano- and micro-scale particles to improve drug permeability, it is crucial to understand how these different types of particles diffuse through the colonic mucus layer.

Investigating drug diffusion or particle movement in a mucus environment ideally requires native mucus samples. However, their use is often constrained by ethical concerns, limited sample availability, and interindividual variability [5]. To address these challenges, artificial mucus models have been developed as cost-effective and standardized alternatives for exploring drug diffusion [6]. These models aim to replicate the key properties of native mucus, though their reliability must first be thoroughly verified. The choice of biopolymers or biomaterials—such as polyacrylic acid (PAA) [6], cellulose [7], sodium hyaluronate [8]—plays a pivotal role in determining how closely artificial mucus mimics the structural and functional characteristics of native mucus. Comparing different mucus models requires analysis of their rheological properties, which directly affect drug transport [9]. Among these properties, the elastic modulus reflects the ability of the mucus hydrogel to recover after stress exposure, while the viscous modulus quantifies its resistance to flow. Another key rheological parameter is creep compliance, which characterizes the time-dependent strain response of a material under a constant load. Mathematically, it is defined as the ratio of shear strain to shear stress and provides insights into the hydrogel’s tendency for permanent deformation [10]. Although these macroscopic rheological properties help establish how mucus behaves in bulk systems, they do not fully reflect the microscopic interactions encountered by drug carriers.

Macrorheology, or bulk rheology, primarily characterizes mucus behavior on a macroscale—such as its role in clearance and lubrication—whereas, microrheology focuses on the viscoelasticity properties of nano- and micro-scale objects [10]. Microrheological studies offer high spatial resolution and can reveal sample heterogeneity that is often overlooked in bulk rheological techniques [10]. As a biopolymer, mucus consists of an intricate network of entangled fibers interspersed with microscopic spaces filled with low-viscosity fluid (herein mainly water). Microrheology enables the precise measurement of the mechanical properties of such soft materials. One commonly used passive microrheology technique is video particle tracking, which relies on the thermal diffusion of particles to assess the heterogeneity of soft materials [11]. By analyzing particle trajectories, this method provides valuable insights into the local mechanical environment within mucus that, for example, drug particles encounter during diffusion.

Understanding nano- and micro-scale particle diffusion within native or artificial mucus matrices is essential for designing drug carriers with enhanced mucus-penetrating properties. Mucus permeability is a complex process influenced by local interactions between particles and the macromolecular matrix formed by mucin, DNA [12], peptides [13], and other biopolymers. The pore size [14] and viscosity [9] of mucus significantly impact the diffusion mechanisms of the particles across various sizes, affecting their transport efficiency. Furthermore, a relationship has been established between apparent permeability and the diffusion coefficient in the mucus environment: a higher diffusion coefficient corresponds to increased permeability, while a thicker mucus layer results in reduced permeability [15]. One of the most widely used techniques for determining nano- and micro-scale particle diffusion is particle tracking, which not only yields diffusion coefficients but also provides insights into additional aspects of particle diffusion behavior, such as diffusion mode types and the microrheological properties of the mucus hydrogel.

Diffusion coefficients provide a fundamental measure of particle mobility; however, they alone are insufficient to fully capture the complexity of particle movement in mucus. To address this limitation, diffusion fingerprinting techniques have been developed, using multiple trajectory-based features to characterize the intricate dynamics of particle transport [16]. This approach extracts features from raw trajectory data, enabling the application of machine learning models or convolutional neural networks for predictive analysis [16–18]. Recently, Pinholt et al. demonstrated the effectiveness of diffusion fingerprinting in distinguishing the movement of nanoparticles with different surface coatings—such as pure polylactic-co-glycolic acid and d-tocopheryl polyethylene glycol 1000 succinate—within mucus layered over a membrane [16].

The features used in previous fingerprinting studies include classical diffusion parameters such as step length distribution, mean square displacement (MSD), diffusion coefficient, and scaling exponent α-values obtained by fitting MSD profiles to a power-law equation. Additional features describing particle motion persistence, confinement, and material heterogeneity—such as kurtosis, fractal dimension, efficiency, trappedness, and Gaussianity—have also been incorporated. However, previous fingerprinting studies have largely overlooked features related to the microrheology of mucus-type hydrogels. Given the complex diffusion patterns of particles in mucus, the characterization of the viscoelastic environment to which these particles are exposed to is essential when optimizing drug delivery strategies.

In this study, we investigated the diffusion patterns of nano- and micro-scale particles with varying sizes and electrostatic states across three mucus types—one native colonic sample of porcine origin and two artificial models—by combining particle tracking with diffusion fingerprinting. Our fingerprinting approach incorporated previously established trajectory-based features, supplemented with descriptors relevant to microrheology. We first assessed the ability of diffusion fingerprinting to differentiate the diffusion properties of the particles across different mucus models. Next, we analyzed the micro-rheological characteristics of these models and compared them with bulk rheological properties. Finally, we identified the artificial mucus model that best replicates the key diffusion behaviors observed in the native system, providing insights into the development of improved mucus-mimicking materials for drug delivery applications.

## Materials and methods

### Materials

Polyacrylic acid (PAA; Carbopol 974P NF) was purchased from Lubrizol (Brussels, Belgium). Hydroxyethylcellulose (HEC; Natrosol 250 HHX PHARM) was a gift from Ashland (Columbus, OH, USA). Porcine mucin type II (from porcine stomach), bovine serum albumin (BSA), cholesterol, Tween 80, 2-(bis(2-hydroxyethyl)amino)ethane sulfonic acid (BES), 2-(N-morpholino)ethanesulfonic acid (MES), MgSO_4_·7H_2_O, and CaCl_2_ were purchased from Sigma-Aldrich (St. Louis, MO, USA). Phosphatidyl choline was procured as a gift from Lipoid GmbH (Ludwigshafen, Germany). The details of artificial mucus formulation can be accessed in our previous study [19]. For negatively charged particles, latex beads (carboxylate-modified polystyrene) with sizes 0.1 (F8803), 0.2 (F8811), and 1 (F8823) µm were bought from Thermo Fisher Scientific and the 0.5 μm (L3280) from Sigma-Aldrich. For the positively charged particles model, latex beads (amine-modified polystyrene) with 0.1 μm mean particle size were procured from Sigma-Aldrich ((L9904), and for 0.2 and 1 μm size from Thermo Fisher Scientific (F8764, F8765). 5 M sodium hydroxide and 5 M hydrochloric acid were used for pH adjustments [19].

### Macrorheology measurement and creep calculation

An ARES-G2 strain-controlled rheometer (TA Instruments, Delaware, USA) equipped with an advanced peltier system accessory was used for rheological measurements. Mucus sample rheology was profiled at 37 °C using a solvent trap to prevent evaporation, following a previously reported protocol [2, 19]. Briefly, elastic and viscous moduli (*G*′ and *G*′′, respectively) were measured using a frequency sweep over at angular frequency of 0.63–60 rad/s at 0.5% oscillation strain. These settings were within the linear viscoelastic region, thereby avoiding structural damage. The rheological profiles of the artificial mucus samples were compared with those of the native ones, as established in a previous study [2, 20]. Creep behavior was calculated from the frequency sweep data using the continuous relaxation time spectrum in the Trios™ software. Creep compliance $\:\left(J\left(\tau\:\right)\right)$ values were obtained by solving the corresponding equation via Laplace transformation [19].

### Image acquisition, preprocessing, and particle tracking with trackpy

Particle movement was investigated using a custom-built spinning disk confocal microscope based on an Eclipse Ti2 body (Nikon), equipped with a 100×/1.42 NA Plan Apo Lambda objective (Nikon). Latex beads were examined in triplicate for each setting. The particles included polystyrene particles with diameters of 100, 200, and 1000 nm (carboxylate modified and hence negatively charged) and 100, 200, and 1000 nm (amine modified and hence positively charged). These were studied in the following models: porcine artificial colonic mucus (PACM) with PAA as the main polymer (PACM-PAA), PACM with hydroxyethyl cellulose (HEC) as the main polymer (PACM-HEC), and porcine native colonic mucus (PNCM). All measurements were performed in triplicate from three different batches of production and three different pigs. The particle sizes fall within the nano- to microscale, corresponding to the ultrafine to fine size range [21]. The suspended latex beads were sonicated following the manufacturer’s instructions, after which 0.3 µL of the particle suspension was mixed with 40 mg of mucus. The dilution volume was minimal (< 1% of the mucus volume) to avoid influencing mucus viscosity. Each time-lapse video was at least 100 s in duration, with images acquired at 1 Hz in triplicate. Samples were incubated in a humidified chamber at 37 °C. To avoid thermal drift, the samples—loaded in in 18-well µ-Slides (Ibidi, Gräfelfing, Germany)—were equilibrated for ∼45 min prior to recording [19, 22].

Fiji, an open source software, was used to preprocess each image frame of the recorded videos [23]. First, a morphological top-hat operation was applied using a circular structuring element of size 10. This step helped isolate small, bright nano and micro-scale particles in the image while filtering out the uneven background caused by refracted light (stemming from the mucus’ complex composition). Next, image contrast was enhanced to improve particle detection. The images were then converted to an 8-bit format to allow for faster processing speed. In total, 131 videos of particle movement, each consisting of 100 frames, were preprocessed (Table S1). Next, particle tracking was performed using TrackPy, a widely used Python library for particle tracking [24]. The “signal” strength and uncertainty estimates were derived based on previous research [25]. For more details of TrackPy analysis, please refer to supplementary section. For details regarding the trajectory data analysis workflow, please see Fig. S15, and for details of video microscopy data, please see Table S1.

### Feature descriptions

Diffusional fingerprinting is a machine learning classification framework that utilizes multi-feature trajectory analysis. Twenty features were selected to characterize the general trends of particle trajectories obtained from the tracking experiments. These features were collected for all tracked particles to account for the effects of varying particle sizes and electrostatic interactions on diffusion within different mucus hydrogels. Fifteen of the selected features were adopted from previous literature. Among them were parameters derived from MSD fitting, including the diffusion coefficient (*D*) and the scaling exponent (*α*). These parameters were obtained by fitting the MSD of each trajectory to a power-law equation: $\:MSD=D{t}^{\alpha\:}$, where *α* is the scaling exponent and corresponds to the slope of the log–log MSD curve. In addition to *D* and *α*, we included the p-value from a chi-squared test to evaluate the goodness of fit, following the approach employed by Pinholt et al. [16].

We also included features that describe the shape and complexity of particle trajectories, such as fractal dimension, efficiency, and trappedness. The fractal dimension quantifies the spatial complexity of a trajectory, while efficiency measures how directly a particle travels from start to finish. Trappedness evaluates the likelihood of a particle being confined within a specific area, reflecting environmental constraints [16–18].

To explore material heterogeneity, we incorporated features such as Gaussianity and kurtosis [16, 18, 26]. Gaussianity assesses deviations from Gaussian motion by comparing higher-order moments of displacement distributions. Kurtosis measures the tailedness of a distribution, which refers to how frequently outliers occur. Specifically, excess kurtosis evaluates the tailedness relative to a normal distribution, offering insights into the likelihood of extreme displacements in the trajectory data.

To capture the general trends in particle speed within the diffusing medium, we included features like the mean step length and mean MSD. These features provide insights into the average displacement per frame and overall diffusion patterns. Following the methodology of Pinholt et al., we also incorporated features derived from Hidden Markov Model (HMM) analysis [16]. These features enabled the classification of trajectories into distinct dynamic states, quantification of the fraction of time spent in each state, and calculation of the average duration of these states.

Finally, we included features associated with the rheological properties calculated from the particle trajectories. These features include creep compliance $\:\left(J\left(\tau\:\right)\right)$, complex shear modulus $\:\left({G}^{*}\right)$, elastic or storage modulus $\:\left({G}^{{\prime\:}}\right),\:$viscous or loss modulus$\:\:\left({G}^{{\prime\:}{\prime\:}}\right)$ and viscosity $\:\left(\eta\:\right)$. Creep compliance quantifies a material’s tendency to deform under applied stress over time. It is directly proportional to the MSD of particle motion and is calculated using the following Eqs. [26, 27]:

1 $$\:J\left(\tau\:\right)=\frac{3\pi\:a}{d{k}_{B}T}\:\langle\Delta\:{r}^{2}\left(\tau\:\right)\rangle$$

where *a* denotes the particle radius (in meters), *d* indicates the dimensionality (two-dimensional (2D) or three-dimensional (3D)), $\:{k}_{B}$ represents the Boltzmann constant, *T* is the temperature (in Kelvin), and $\langle\Delta\ {r}^{2}\left(\tau\:\right)\rangle$ signifies the time-averaged MSD at lag time $\:\tau\:$. The average creep compliance was calculated by averaging $\:J\left(\tau\:\right)$ over all lag times.

The complex shear modulus $\:{(G}^{*})$ characterizes the overall viscoelastic response of the material by combining both the storage modulus $\:\left({G}^{{\prime\:}}\right)$ and viscous modulus $\:\left({G}^{{\prime\:}{\prime\:}}\right)$. Using the Laplace transform approach, $\:{G}^{*}$ was calculated as

2 $$\:{G}^{\text{*}}=\frac{d{k}_{B}T}{3\pi\:a \langle Delta\:{r}^{2}\left({t}_{o}\right)\rangle\:\varGamma\:\left[\alpha\:\left({t}_{o}\right)+1\right]},$$

where $\Delta\:\langle{r}^{2}\left({t}_{o}\right)\rangle$is the MSD at the characteristic time $\:{t}_{o}=\frac{1}{{s}_{o}}$, and *α* is the scaling exponent (also referred to as the critical relaxation exponent in the Laplace-domain generalized Stokes–Einstein relation), obtained as the slope of the log-log MSD curve. *Γ*[*α* + 1] represents the gamma function evaluated at *α* + 1 [28]. A range of Laplace frequencies $\:{s}_{o}\in\:\left[0.01,\:100\right]\:{s}^{-1}\:$was used to capture the viscoelastic behavior across multiple timescales, and the average of $\:{G}^{*}$over the range was reported as the complex shear modulus.

The storage $\:\left({G}^{{\prime\:}}\right)$ and viscous moduli $\:\left({G}^{{\prime\:}{\prime\:}}\right)$ describe the solid-like (elastic) and liquid-like (viscous) behavior of the material, respectively. These values were derived from angular frequency-dependent $\:{G}^{*}$ as [28]

3 $$\:{G}^{{\prime\:}}\left(\omega\:\right)=\:\left|{G}^{\text{*}}\right|cos\left(\pi\:\alpha\:/2\right),\:{G}^{{\prime\:}{\prime\:}}\left(\omega\:\right)=\:\left|{G}^{\text{*}}\right|sin\left(\pi\:\alpha\:/2\right)$$

where *ω* denotes the angular frequency and *α* is the critical relaxation exponent.

The average $\:{G}^{{\prime\:}}$ and $\:{G}^{{\prime\:}{\prime\:}}$ values were calculated by taking the mean of $\:{G}^{{\prime\:}}\left(\omega\:\right)$ and $\:{G}^{{\prime\:}{\prime\:}}\left(\omega\:\right)$, respectively, over a range of frequencies.

Viscosity *η* was estimated as the ratio of $\:{G}^{{\prime\:}{\prime\:}}\left(\omega\:\right)$ to *ω* in the low-frequency limit:

4 $$\:{\eta\:=\:G}^{{\prime\:}{\prime\:}}\left(\omega\:\right)/\omega\:$$

The average viscosity was computed by averaging *η* over a defined range of frequencies.

By combining these features, our analysis allows the detailed characterization of the microrheology of the system, capturing hows particles perceive and respond to the rheological properties of the materials through which they diffuse.

### Data categorization

The features described in the previous section were calculated for all particle trajectories obtained using TrackPy. To enable detailed analysis, the data were categorized into nine distinct groups (Table 1). Each category includes particle trajectories recorded in the three media described in ''Image acquisition, preprocessing, and particle tracking with trackpy''. The total number of particle trajectories per category is also presented in the Supplementary Table S1.

**Table 1:** Overview of data categories based on particle types used in feature calculation

Dataset name	Particle charge	Particle size (nm)
All Data	Positive, Negative	100, 200, 1000
Positively charged	Positive	100, 200, 1000
Negatively charged	Negative	100, 200, 1000
Positively charged 100 nm	Positive	100
Negatively charged 100 nm	Negative	100
Positively charged 200 nm	Positive	200
Negatively charged 200 nm	Negative	200
Positively charged 1000 nm	Positive	1000
Negatively charged 1000 nm	Negative	1000

### Machine learning models

Following feature extraction and data categorization, all datasets were standardized feature-wise to have a mean of zero and a variance of one, ensuring equal weight across. To evaluate the effectiveness of the diffusional fingerprinting approach, we employed a variety of classifiers and assessed their ability to identify the materials in which particles were diffusing. The models used included support vector machines (SVMs) with radial basis function (RBF) kernel, k-nearest neighbors (kNN), histogram-based gradient boosting, decision trees, logistic regression, random forest (RF), and Fisher’s linear discriminant. Algorithms were selected based on their classification into three families—linear models, instance-based learners, and ensemble methods. Linear models use a linear relationship between predictors and the output; in this study, we employ logistic regression and Fisher’s linear discriminant analysis. Instance-based learners or memory-based methods classify new samples by comparing them directly to stored examples; here we use kNN and SVM with an RBF kernel. Ensemble or tree-based methods combine multiple base models to improve robustness and capture complex, nonlinear patterns; we evaluate decision trees, RF, and histogram-based gradient boosting. These methods have also been successfully applied to nanoparticle synthesis, nano–bio interaction studies, and various nanotheranostic applications [29].

For all datasets, classification models were trained on a single train–test split (70% training, 30% validation). Additionally, fivefold cross-validation (80% training, 20% validation per fold) was performed for model evaluation. For each fold, overall accuracy, precision, recall, and the F1-score—which treats each class equally and balances precision and recall across classes—ensuring an unbiased performance assessment even in the presence of class imbalance, was calculated. The density distributions for the features across different datasets and classes were computed using kernel density estimation (KDE) with the “kdeplot” function from the Seaborn Python library.

Feature-wise class similarities were calculated using four different metrics: area overlap, Kullback–Leibler (KL) divergence, cosine similarity, and Euclidean distance. The area overlap metric quantifies the shared region under the KDE curves of a given feature across different classes. It reflects the similarity between distributions in terms of range and shape, with higher overlap values indicating greater similarity between classes. The cosine similarity measures the cosine of the angle between two vectors, treating feature histograms as vectors in high-dimensional space [30]. It captures the similarity in distribution orientation, with values closer to 1 indicating greater alignment. The KL divergence measures how one probability distribution differs from another. It quantifies the expected logarithmic difference between the probability values of the two distributions [31]. Finally, the Euclidean distance calculates the straight-line distance between the histograms of two distributions [32]. Smaller distances indicate greater similarity, as they imply that the distributions are closer in overall shape and position.

We also used SHapley Additive exPlanations (SHAP) to interpret the outputs of a “HistGradientBoostingClassifier” trained on scaled feature data for a multiclass classification task. Class-specific SHAP values were computed using the “TreeExplainer.” To quantify feature importance for each class, mean absolute SHAP values were calculated across all samples.

## Results

### Video microscopy to characterize nano- and micro-scale particle movement in mucus

**Fig. 1:**
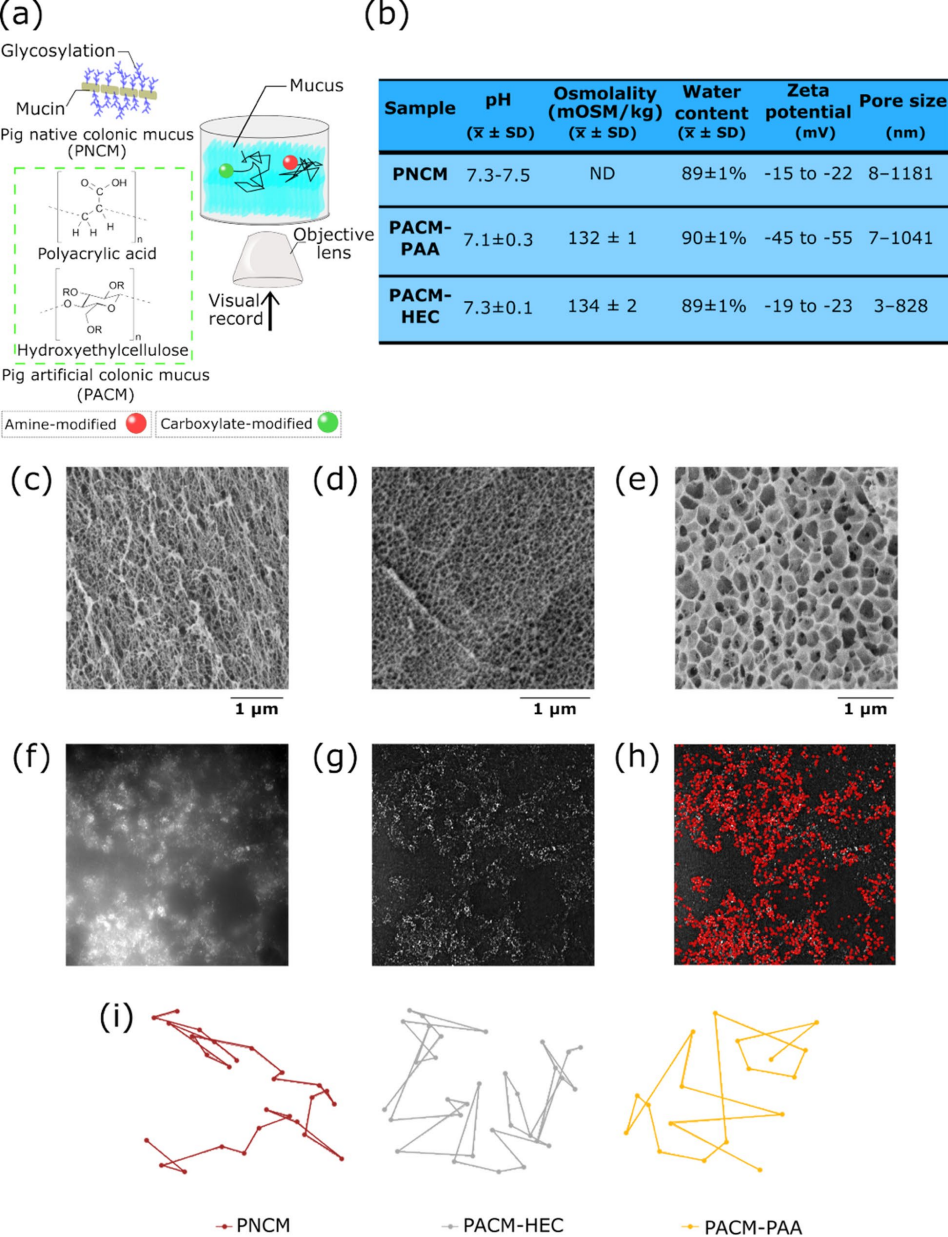
Particle tracking setup for analyzing nano- and micro-scale particle movement in three types of mucus. (**a**) Video microscopy of particle movement in mucus. (**b**) Summary of key physicochemical properties of the three mucus types from previous study [19]. Cryo-SEM pore micrograph of (**c**) PNCM, (**d**) PACM-PAA, and (**e**) PACM-HEC. (**f**) Image preprocessing process showing original microscopy image with strong light refraction; (**g**) after application of top-hat filtering to enhance contrast; and subsequent (**h**) detection and tracking of particles. (**i**) Representative trajectories in the three different mucus models. Abbreviation ND - not determined, used in figure **b**. During the isolation process of the native colonic mucus, ice-cold isotonic buffer was used to keep the mucus hydrated; therefore, a “true value” of the native mucus osmolality might be influenced by the buffer and was not determined [33]

To investigate particle movement in mucus, three types of mucus were studied for their role as diffusion barriers to nanoparticles: PNCM, PACM-HEC, and PACM-PAA. Six types of polystyrene nanoparticles (Table S2) were used to evaluate their diffusional behavior in these matrices. The particles varied in size (100, 200, and 1000 nm) and surface charge, with carboxylate (negatively charged) and amine (positively charged) modifications (Fig. 1a). The results of mucus characterization, performed based on a previous study by Tjakra et al. [19], are summarized in Fig. 1b, highlighting key physicochemical properties influencing particle diffusion. Structural features of the mucus matrices were examined using cryogenic scanning electron microscopy (Cryo-SEM), revealing similar porous structures in PNCM (Fig. 1c), PACM-PAA (Fig. 1d), and PACM-HEC (Fig. 1e).

Video microscopy images (Fig. 1f) were preprocessed using the FIJI software to reduce background noise arising from the complex mucus matrix and particle fluorophores. A “top-hat” filter was applied (Fig. 1g) to enhance image contrast, enabling precise localization of each particle in every frame (Fig. 1h). Finally, representative trajectories were extracted (Fig. 1i), showcasing distinct intra- and inter-mucus movement patterns. The resulting trajectories were recorded in tabular format, with *x* and *y* coordinates of each particle tracked over the time.

### Gradient boosting achieves the highest accuracy in the diffusional fingerprinting model

**Fig. 2:**
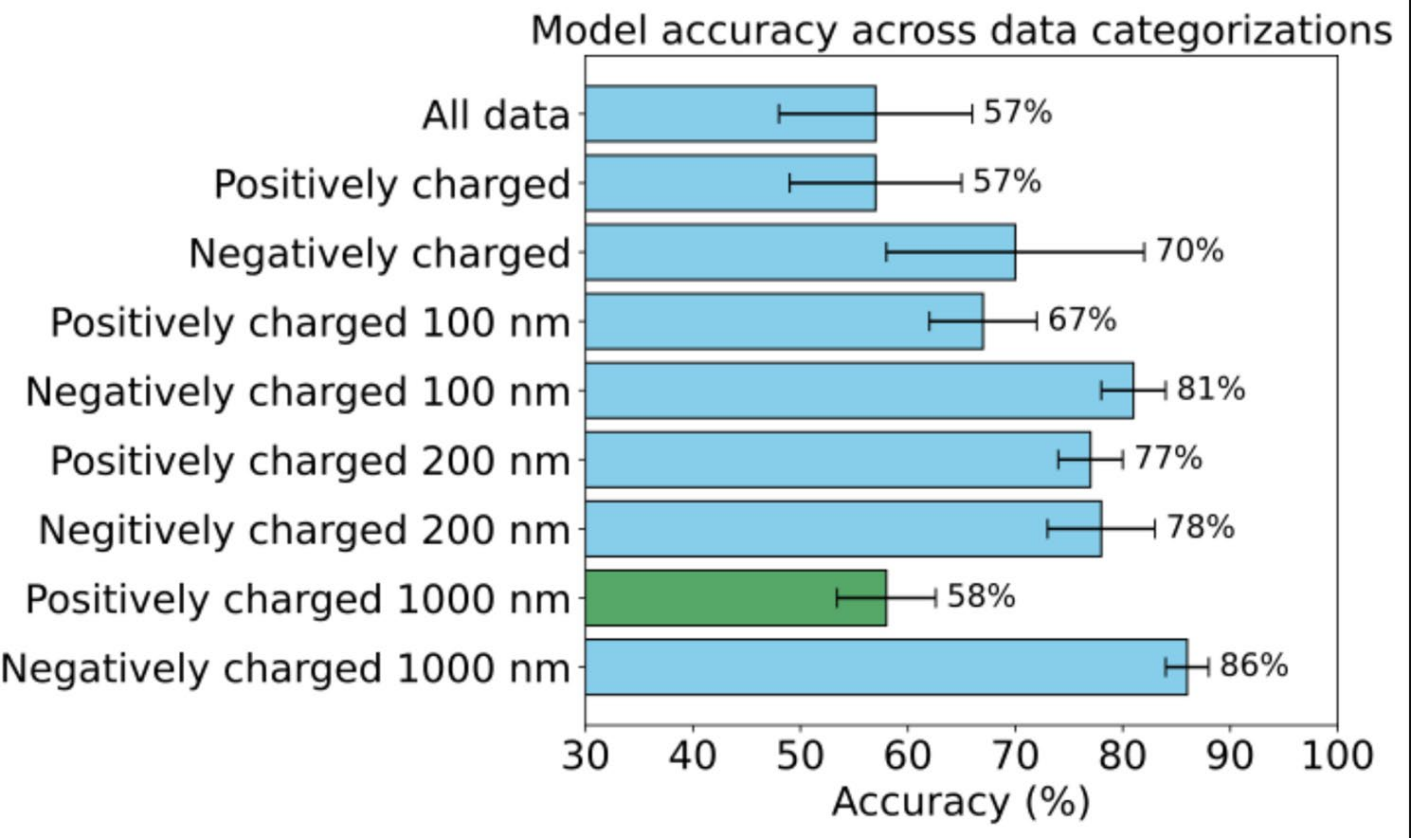
Highest accuracy achieved by different classifiers across data categories. Error bars represent the standard deviation across validation runs. F1 scores for seven different classifiers were evaluated for each data category, as described in the Methods section. Blue bars indicate the highest accuracy achieved using the histogram-based gradient boosting model, while green bars represent the highest performance from the RF classifier

We applied seven different classifiers, as described in the Methods section, and evaluated their performance using F1-scores across all data categories. A high F1-score indicates that the model effectively captures true positives, while minimizing both false positives and false negatives. The highest accuracy achieved for each category is presented in Fig. 2, while detailed F1-scores for all classifiers and datasets are provided in Supplementary Fig. S1. For most datasets, the highest classification accuracy was obtained using the histogram-based gradient boosting model, with the RF model closely following, showing only a 1–2% difference in performance. An exception was observed for the dataset involving positively charged particles (1000 nm), where the RF model achieved the highest accuracy. Notably, Pinholt et al. previously demonstrated that logistic regression achieved higher accuracy in identifying diffusing particles in both binary and multiclass classification tasks using simulated data. However, in our study, logistic regression performed less effectively than histogram-based gradient boosting and RF models in identifying the type of mucus hydrogels from particle tracking data of nano- and micro-scale particles varying in size and charge. This discrepancy likely reflects the high structural heterogeneity of mucus models, which favors tree-based methods like histogram-based gradient boosting. These models are well-suited for capturing nonlinear patterns and effectively handling high-dimensional, sparse data [34, 35].

Figure 2 also reveals that the classification accuracy for the “All Data” and “Positively Charged” categories was relatively low (only 57%). In contrast, accuracy improved substantially when datasets were grouped by specific particle sizes and charges. For negatively charged particles, accuracy reached up to 86%, while for positively charged particles, accuracy improved for certain datasets, peaking at 77%. However, for the “Positively Charged 1000 nm” data category, accuracy remained notably low, even when categorized by both size and charge.

Furthermore, we quantitatively compared the framework proposed in this study, which includes rheological features, with two of the previous classification studies, where no rheological features [16] and no HMM-based and rheological features were included [18]. We compared the F1-accuracy using histogram-based gradient boosting across various datasets employed in this study. The results are summarized in Table S3. It was found that the current study, utilizing both HMM-based and rheological features in addition to conventional descriptors, consistently achieves superior or equivalent F1-accuracy compared to previous classification studies. Particularly notable improvements are observed for positively charged particles of 100 nm (increase from 59 to 67%) and negatively charged particles of 1000 nm (increase from 80 to 86%). The inclusion of HMM-based features introduced by Pinholt et al. could improve accuracy for negatively charged particles of 1000 nm (from 80 to 85%). However, the improvement for positively charged particles of 100 nm was modest, increasing only up to 61%. Even in cases where the differences were modest, the inclusion of rheological features did not negatively impact model performance. This highlights the benefit of integrating rheological descriptors with previously used features to enhance classification accuracy and enable more in-depth material characterization.

**Fig. 3:**
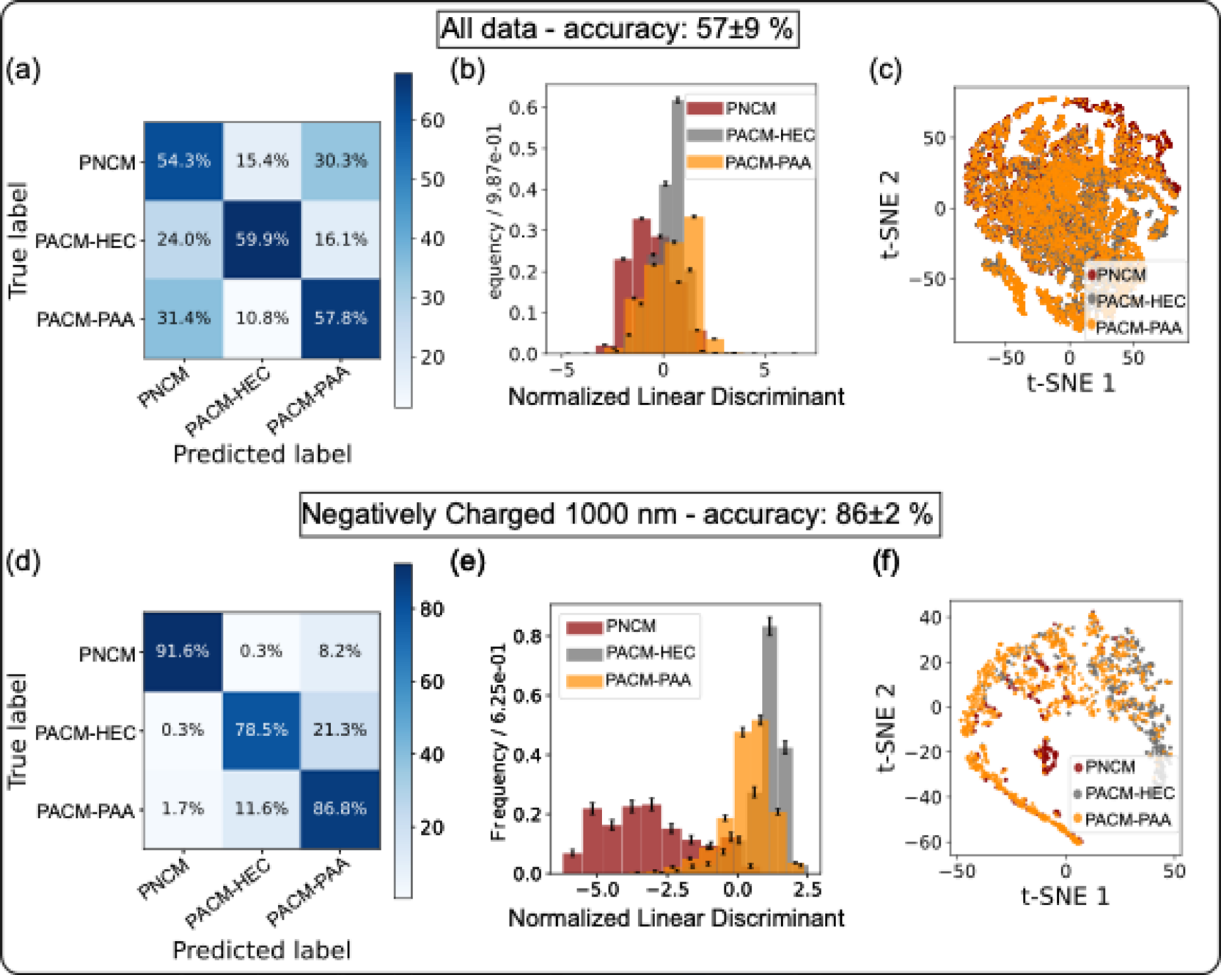
Classification performance comparison for different datasets. (**a**) Confusion matrix for the “All Data” category dataset, with an average accuracy, precision, recall, and F1-score of 57 ± 9%, 57 ± 9%, 59 ± 9%, 57 ± 9%, respectively. (**b**) Normalized linear discriminant distribution for PNCM, PACM-HEC, and PACM-PAA classes. (**c**) t-SNE visualization of the “All Data” category dataset. (**d**) Confusion matrix for the “Negatively Charged 1000 nm” category dataset, with an average accuracy, precision, recall, and F1-score of 85 ± 2%, 87 ± 2%, 86 ± 1%, 86 ± 2%, respectively. (**e**) Normalized linear discriminant distribution for the “Negatively Charged 1000 nm” category dataset. (**f**) t-SNE visualization of the “Negatively Charged 1000 nm” category dataset

To assess classification performance, confusion matrices were generated using fivefold cross validation on the test data following training with the “HistGradientBoostingClassifier.” Figs. 3(a) and (d) show the results for the “All data” category (lowest accuracy) and “Negatively charged 1000 nm” category (highest accuracy). Classification accuracies for the “All data” category was 54.3% (PNCM), 59.9% (PACM-HEC), and 57.8% (PACM-PAA). In contrast, for the “Negatively Charged (1000 nm)” category, accuracies were significantly higher, reaching 91.6% (PNCM), 78.5% (PACM-HEC), and 86.8% (PACM-PAA).

To visualize class separability within the datasets, linear discriminant analysis (LDA) was applied to reduce the 20 diffusional features to a single dimension. In the “All data” category (Fig. 3(b)), the three classes showcased minimal separability, while in the “Negatively charged 1000 nm” category (Fig. 3(d)), distinct class separation was observed with only minor overlap. Further dimensional reduction using t-distributed stochastic neighbor embedding (t-SNE) analysis projected the data into a 2D space (Figs. 3(c) and (f)), reinforcing the LDA findings. The t-SNE plot for “All data” revealed substantial class overlap, while the “Negatively charged 1000 nm” dataset showcased improved class separation. These findings demonstrate the potential of diffusional fingerprinting for accurately classifying nano- and micro-scale particle diffusion and interactions in specific hydrogels, particularly when particle size and surface charge are well defined.

### Nano- and micro-scale particle diffusion modes in native and artificial mucus are influenced by particle size and charge

**Fig. 4:**
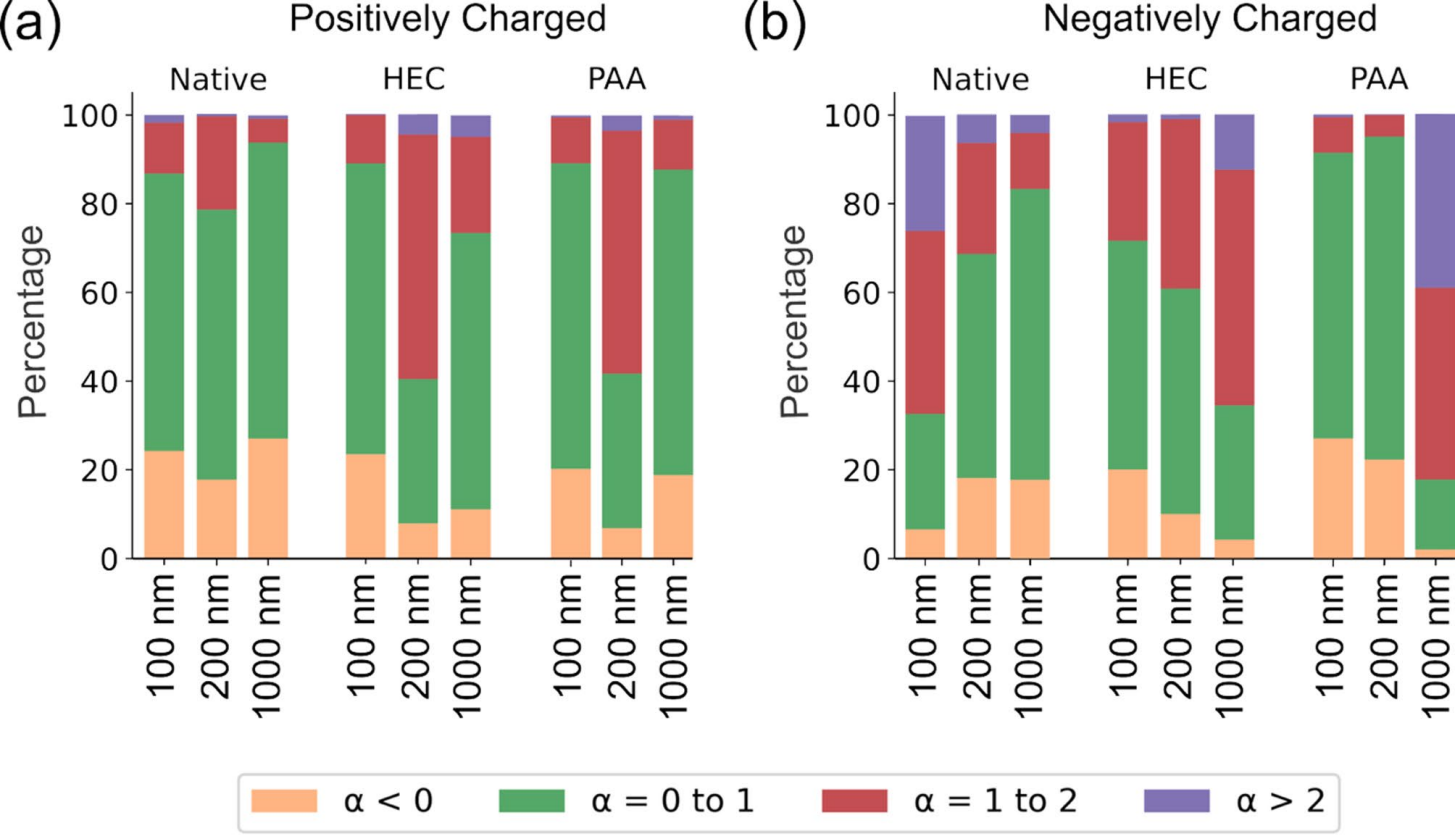
Diffusion modes classified based on α values from MSD power-law fitting of particle trajectories. Panels (**a**) and (**b**) show results for positively and negatively charged particles, respectively, across three mucus hydrogels and three particle sizes. This figure presents stacked bar plots illustrating the percentage of diffusion modes classified by exponent α in the MSD power-law fitting equation. The classification includes immobile (α < 0, orange), subdiffusive (0 ≤ α < 1, green), diffusive (1 ≤ α ≤ 2, red), and active (α > 2, purple)

Before analyzing the various factors affecting particle diffusion, we first examined the dominant diffusion modes across the three different hydrogels. Using datasets of particles with specific sizes and charges, we categorized their diffusion based on the scaling exponent (obtained by fitting the MSD of each trajectory), *α* values: *α* < 0 (immobile), 0 ≤ *α* < 1 (subdiffusive), 1 ≤ *α* < 2 (diffusive), and *α* > 2 (active diffusion). Figure 4 illustrates that most particles exhibited immobile or subdiffusive behavior, suggesting confined particle movement and matrix heterogeneity [16, 24]. Positively charged nano- and micro-scale particles (100 and 1000 nm) showed the highest immobile (*α* < 0) and subdiffusive (*α* < 1) percentages (85–90%) across all three mucus matrices, leading to reduced fingerprinting accuracies of 67% and 58%, respectively (Fig. 4a). Their restricted movement likely results from aggregation and interaction with the mucus macromolecules, given their strong interactions with negatively charged mucus components [5, 25]. Interestingly, Fig. 4a also shows that 200 nm positively charged nanoparticles displayed reduced confinement in artificial mucus (PACM-HEC and PACM-PAA), indicating that electrostatic interactions, rather than size alone, influence mobility.

For negatively charged particles, Fig. 4b shows that the percentage of particles in the immobile and subdiffusive states increased from approximately 35% (100 nm) to 85% (1000 nm) in PNCM. However, in PACM-PAA, 100 and 200 nm particles remained largely immobile (> 90%), while 1000 nm particles exhibited higher mobility. Among particles in the artificial mucus, PACM-HEC demonstrated diffusion behavior most similar to native mucus.

To further elucidate the size- and charge-dependent movement of the particles, we plotted the distribution of fractal dimensions for six data sets consisting of particles with specific sizes and charges (see Supplementary Figure S2). The fractal dimension is a measure of how space-filling a particle’s trajectory is. It is approximately 1 for straight, ballistic motion, around 2 for normal Brownian diffusion, and greater than 2 for subdiffusive or confined motion. Supplementary Figure S2 shows that negatively charged 1000 nm particles exhibit peaks near 2, indicating relatively unconfined Brownian motion. In contrast, the two positively charged particles (100 nm and 1000 nm) show peaks furthest from 2, suggesting subdiffusion and more confined movement [16]. This suggests that positively charged particles, particularly those smaller or larger than a certain size, experience the most restricted motion, which is consistent with the diffusion states estimated from the alpha values.

In summary, nano- and micro-scale particle diffusion within mucus is highly constrained, influenced by both size and electrostatic interactions. These findings underscore the importance of considering multiple factors when evaluating particle transport in biological and artificial mucus environments.

### SHAP analysis highlights key features in distinguishing particle diffusion across different mucus

**Fig. 5:**
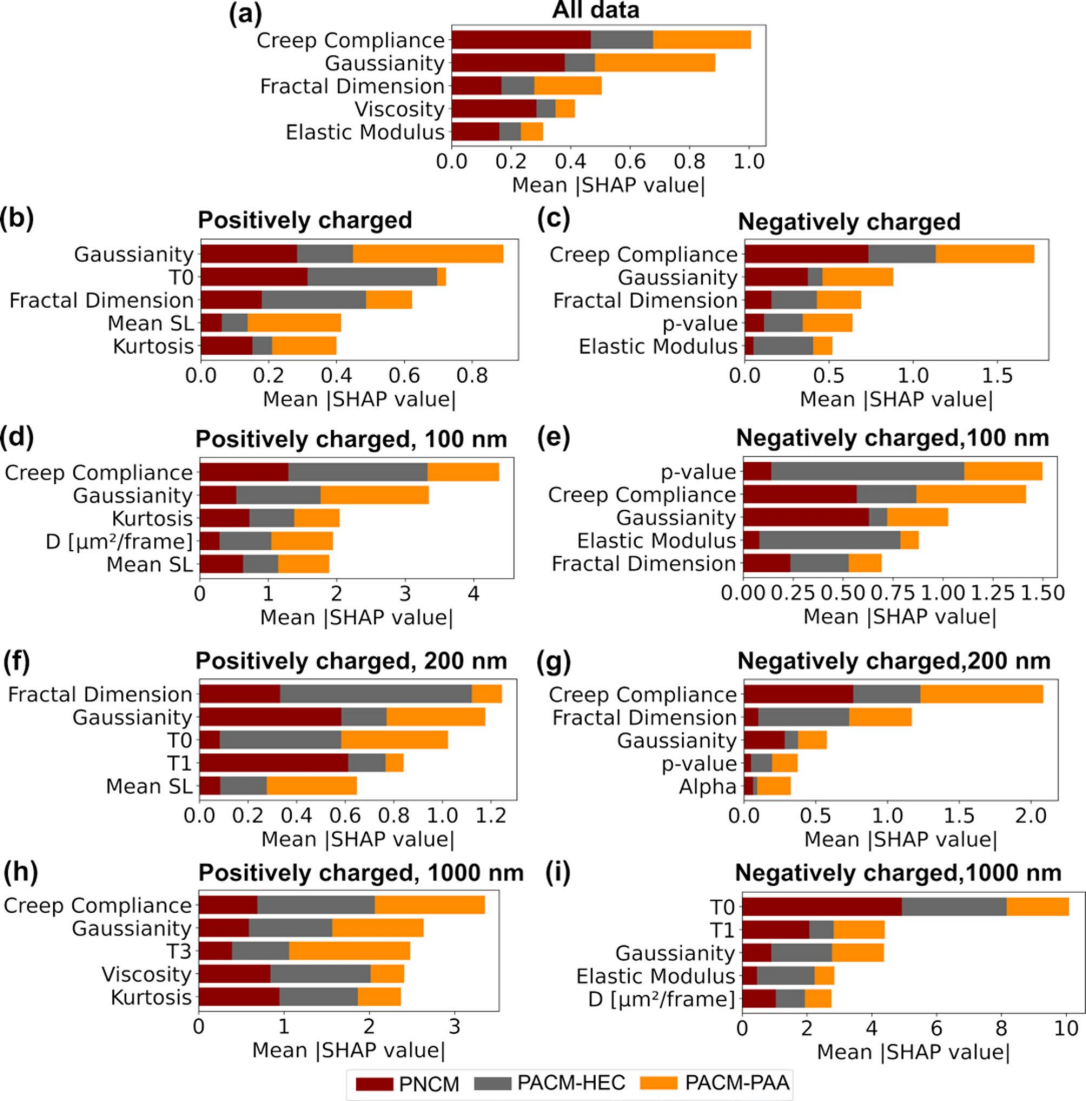
Top five features based on class-wise SHAP feature importance for classifying PNCM, PACM-HEC, and PACM-PAA across all datasets. A multiclass model was trained on scaled features with numerically encoded labels. Class-specific SHAP values were computed using “TreeExplainer” and averaged (absolute) across samples to obtain feature importance. The results are visualized as a stacked bar chart, showing the relative importance of each feature across the three classes. Abbreviations: Mean SL- mean step length, T0, T1, and T3 are features derived from Hidden Markov Model (HMM) analysis

We analyzed the density distribution of each feature across different classes within various data categories. The density distributions of 20 features across nine data categories are provided in Supplementary Figs. S3–S11. The analysis reveals that while some features clearly differ across classes, others are more difficult to distinguish. To identify the features contributing most to class predictions, we used SHAP values from a trained machine learning model. Figure 5 displays the top five features ranked by total SHAP importance across all classes. Each horizontal bar is divided into segments representing the contribution of that feature to each class, allowing the comparison of class-wise importance within each feature.

In the category with the highest F1-accuracy (Negatively Charged, 1000 nm), HMM-based features played a significant role in distinguishing the classes (Fig. 5i). Features related to material heterogeneity—such as Gaussianity, which measures how closely the displacement distribution follows a normal (Gaussian) distribution—and rheological properties—including elastic modulus and viscosity—were also among the top contributors. Across most datasets, features derived from rheological properties, material heterogeneity, and trajectory shape were the most influential. Specifically, creep compliance (a rheological-property-based feature) emerged as the top feature in several datasets (Fig. 5a, c, d, g, and h), many of which exhibited high F1 scores (> 78%).

As described in Eq. 1, creep compliance is proportional to the mean squared displacement (MSD) of particle motion and reflects the material’s deformability. In more deformable materials, particles tend to move more over time. While macroscopic properties such as water content and pore size may appear similar between different materials (Fig. 1), micro-scale deformability can still vary due to differences in material composition—for example, the presence of mucin glycosylation or variations in polymer backbones between artificial mucus systems. Additionally, the physicochemical properties of the particles themselves—such as size, shape, and surface charge—can influence how effectively they deform the surrounding material. Although rheological features were not explicitly included in previous classification studies, Pinholt et al. incorporated the mean MSD as one of the features in their analysis [16]. They identified it as a key factor in distinguishing particle types diffusing on a mucus model layered over a lipid membrane.

Other rheological features, such as elastic modulus, viscous modulus, and viscosity, also ranked among the top five features for several data categories (Fig. 5a, c, e, and h). The density distribution of these rheological features varied distinctly across different hydrogels within each category. These variations in rheological feature distributions made them highly discriminative for diffusion across the matrices, ranking them among the top features (see Supplementary Figs. S3–S11). Overall, Fig. 5 suggests that the inclusion of new rheological-property-based features improved the identification of particles within specific mucus. These features highlighted differences in the microrheology of the environments through which the particles were moving.

### Rheological properties calculated from particle trajectories differ from bulk experimental measurements

**Fig. 6:**
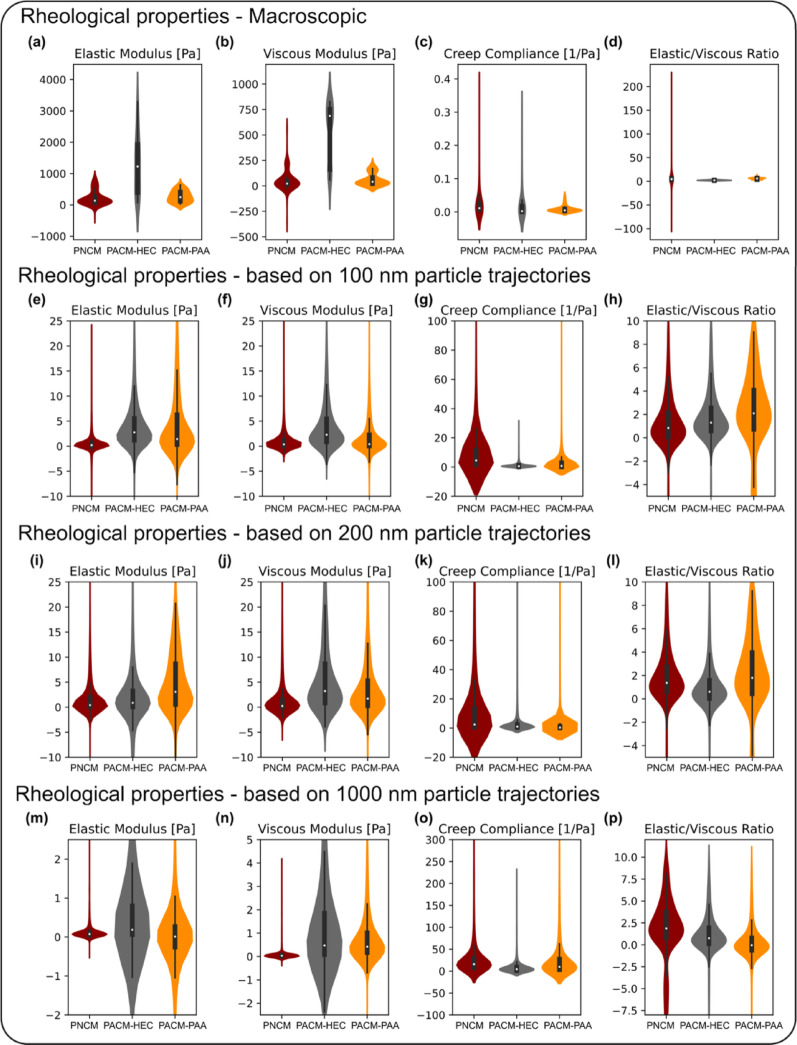
Comparative analysis of rheological properties–including elastic modulus, viscous modulus, viscosity, and creep compliance—across different mucus types: PNCM, PACM-HEC, and PACM-PAA. Violin plots combined with box plots were used to visualize the data. The first row (**a**–**d**) displays experimental bulk measurements, while the second (**e**–**h**), third (**i**–**l**), and fourth rows (**m**–**p**) correspond to values calculated from particle trajectories of 100, 200, and 1000 nm particles, respectively. Each plot combines features of a box plot and a kernel density plot. The white dot at the center of each violin plot represents the median value. The box surrounding the dot indicates the interquartile range, covering the middle 50% of the data. The lines extending from the box represent the data range, excluding outliers, and help visualize overall data spread and variability. The outer violin shape illustrates the full distribution density, showing where data points are more concentrated or spread out

We further investigated the experimental bulk rheological properties of different mucus compositions and compared them with the rheological properties derived from particle trajectories. Figure 6 presents the rheological properties obtained from experimental measurements and from particle trajectories of 100, 200, and 1000 nm particles, respectively, using violin plots combined with box plots. Based on the analysis, it is evident that both bulk elastic and viscous moduli obtained from experiments are significantly higher than those derived from particle-trajectory-based calculations. This suggests that the microscale environment exhibits lower resistance to deformation than what is observed in bulk rheology measurements. In contrast, creep compliance follows an inverse trend—experimental data show lower compliance (indicating higher resistance to deformation), whereas trajectory-based calculations yield higher compliance values.

For the experimental bulk properties (Figs. 6a–d), PACM-HEC exhibited a broader distribution compared to PNCM and PACM-PAA. Except for creep compliance, the distributions of PNCM and PACM-PAA were very similar. The median elastic and viscous moduli of PACM-HEC were significantly higher than those of PNCM and PACM-PAA. For all four properties, the median values of PACM-PAA were closer to those of PNCM.

For the particle-trajectory-based calculations, the median values of all four rheological properties were similar across the three mucus types. However, PNCM exhibited a distinct distribution pattern in relation to PACM-HEC and PACM-PAA (Figs. 6e–p). PNCM showed the lowest mean elastic and viscous moduli, across all three particle sizes, while PACM-HEC and PACM-PAA exhibited higher values. Additionally, the distributions of elastic modulus, viscous modulus, and viscosity in the PNCM medium were narrower, whereas PACM-HEC and PACM-PAA displayed broader distributions. For creep compliance (Figs. 6h, l, and p), the PNCM medium displayed a wide compliance range, indicating higher deformability than those of PACM-HEC and PACM-PAA. Moreover, the distribution of elastic modulus, viscous modulus, and creep compliance for the PNCM system with 1000-nm particles was found to be relatively compact. This suggests that large particles experience a more homogeneous environment compared to small particles. We also calculated the ratio of elastic modulus to viscous modulus and observed an increasing trend for the PNCM system. Interestingly, this ratio is > 1 for 40%, 60%, and 68% of the 100, 200, and 1000 nm particles, respectively. This suggests that, in the PNCM system, large particles interact strongly with the elastic part of the network, while smaller particles primarily experience the viscous component. This stronger interaction with the elastic component of the network increases the proportion of particles in the subdiffusive state for larger particles, as shown in Fig. 4.

### Class similarity analysis identifies similarities in particle diffusion across different mucus types

**Fig. 7:**
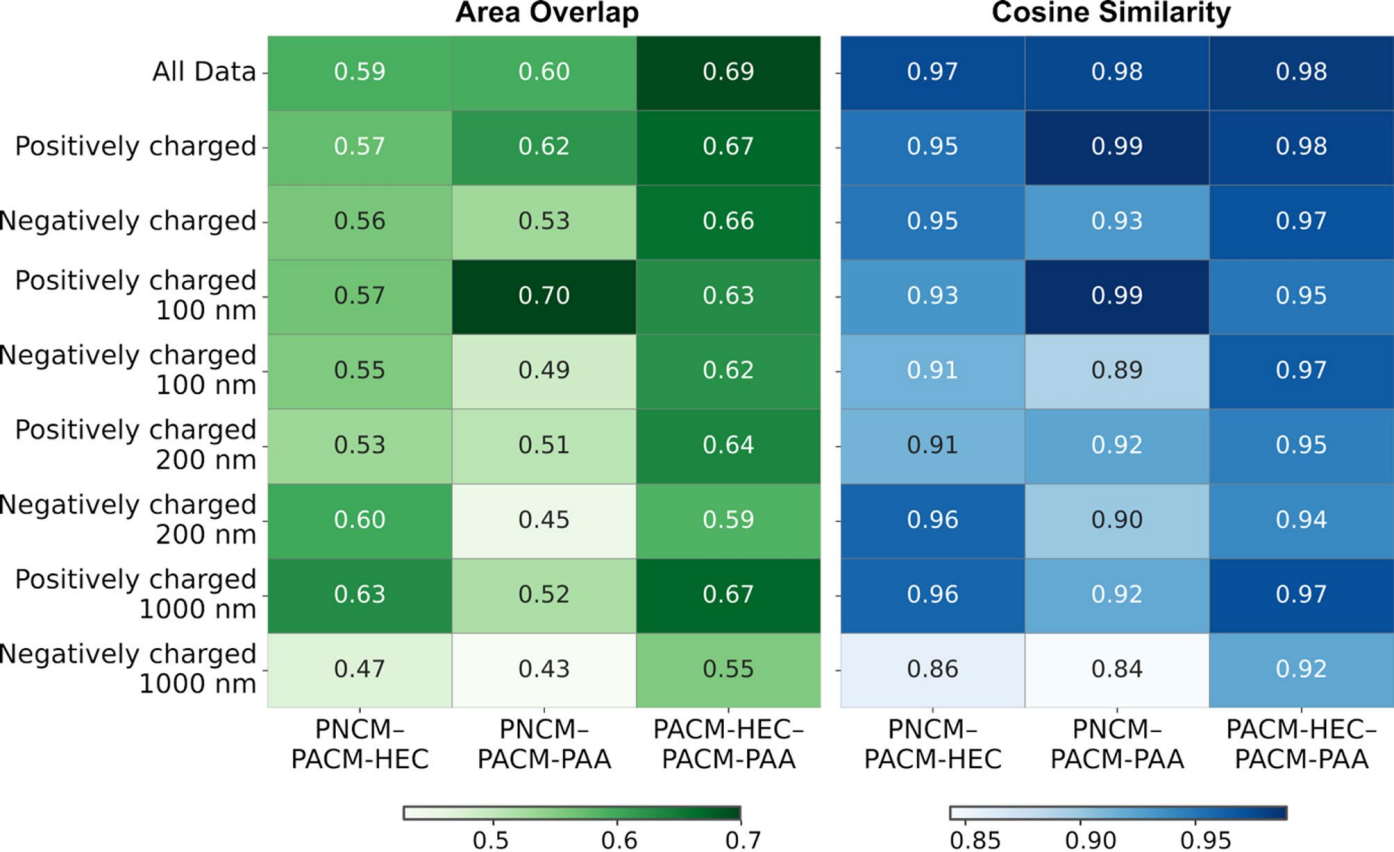
Comparison of similarity metrics for pairwise comparisons among the three mucus models—PNCM, PACM-HEC, and PACM-PAA—using area overlap and cosine similarity across all datasets. The values represent the mean similarity scores calculated across all 20 features, providing a measure of central tendency

We also investigated feature-wise class similarities by analyzing the distribution of each feature using four different metrics: area overlap, KL divergence, cosine similarity, and Euclidean distance (see Methods). Each metric was computed for all 20 features, providing a comprehensive comparison of feature distributions across classes. Figure 7 summarizes the mean similarity values using area overlap and cosine similarity. While Fig. 7 shows only the means, detailed box plots for area overlap and cosine similarity are in Supplementary Figure S13, and those for KL divergence and Euclidean distance in Figure S14, highlighting the variation and central tendency across features and data categories.

Figure 7 shows that, according to the area overlap metric, the mean value for PACM-HEC–PACM-PAA comparison is the highest across most datasets. This suggests that PACM-HEC and PACM-PAA share more similar feature distributions than either does with PNCM. The PNCM–PACM-HEC mean area overlap value is consistently higher than that of PNCM–PACM-PAA across all datasets derived from experiments using negatively charged nano- and micro-scale particles and in two datasets pertaining to positively charged particles (200 and 1000 nm). For cosine similarity, Fig. 7 reveals that the mean values for all three pairwise comparisons were higher than 0.9, indicating high overall similarity. However, the PACM-HEC–PACM-PAA comparison exhibited the narrowest distribution across most datasets (see Figure S13). Similar to the area overlap results, the PNCM–PACM-HEC showed a narrower distribution and higher mean values across features compared to the PNCM–PACM-PAA comparison across all data, including those with negatively charged particles and the 1000 nm positively charged particle dataset, as shown in Fig. 7 and S13. These findings suggest that particle diffusion in the two artificial mucus models is relatively similar. Moreover, the diffusion behavior of negatively charged particles in PACM-HEC more closely resembles that in PNCM than in the PACM-PAA system.

To further elucidate which features drive the observed similarities, we examined the feature-wise area-overlap metrics in more detail. For every feature, we calculated the difference between the PNCM–PACM-HEC and PNCM–PACM-PAA area-overlap values; a larger positive difference indicates that the feature contributes more strongly to the PNCM–PACM-HEC similarity. The five features with the largest positive differences in each dataset (grouped by particle size and surface charge) are presented in Supplementary Fig. S12. Notably, viscosity emerged as the dominant contributor in four datasets—negatively charged 100 nm and 200 nm particles, and positively charged 200 nm and 1000 nm particles. A high viscosity overlap indicates that the shear resistance experienced by particles in PACM-HEC closely matches that in PNCM, implying comparable hydration and network porosity. In the positively charged 100 nm and negatively charged 1000 nm systems, Gaussianity was the principal contributor. Because Gaussianity quantifies how closely the particle step-length distribution follows a normal profile—and thus reflects microstructural heterogeneity—its prominence here suggests that particles in PACM-HEC encounter a heterogeneity landscape similar to that in PNCM under these specific conditions.

The KL divergence and Euclidean distance metrics followed a similar trend, with the PACM-HEC–PACM-PAA comparison showing the lowest divergence and distance values, further reinforcing the similarity between these two mucus (Supplementary Figure S14). However, no specific trend was observed between PNCM–PACM-HEC and PNCM–PACM-PAA. In general, the median divergence and distance values were close to zero for both PNCM–PACM-HEC and PNCM–PACM-PAA across all datasets, although the breadth of their distributions varied depending on the dataset.

## Discussion

### Diffusional fingerprinting enables the identification of negatively charged nano- and micro-scale particle diffusion across various mucus compositions

The findings of this study indicate that the accuracy of diffusional fingerprinting for identifying nano-and micro-scale particle diffusion in both native and artificial mucus matrices is influenced by dataset composition, such as the inclusion of trajectories obtained from particles of different sizes and charges. When the dataset contained particles of various sizes and charges, classification accuracy was relatively low (only 57%). Grouping the dataset based on particle charge alone improved accuracy for negatively charged particles (70%), while no improvement was observed for positively charged particles. Further grouping the dataset based on both charge and size led to a significant increase in accuracy, reaching 78–86% for datasets containing particles with a specific charge and size.

The highest classification accuracy was observed for the negatively charged 1000-nm particles. This dataset exhibited the strongest similarity to normal diffusion behavior, as indicated by multiple features, including alpha values and fractal dimension. Specifically, the alpha values were greater than 1, and the fractal dimension distribution peaked near 2—both hallmarks of unconfined Brownian motion. Notably, the size of these particles closely matched the characteristic length scale or average pore size of the material (approximately 1000 nm; see Fig. 1). This size compatibility likely enabled the particles to diffuse more freely through the porous network, minimizing confinement effects and resulting in motion that closely resembles normal diffusion. Among positively charged particles, only the 200-nm-diameter group exhibited accuracy levels comparable to those of the negatively charged particles.

These findings indicate that the negatively charged particles exhibit a distinct diffusion behavior across the examined mucus hydrogels, largely independent of particle size. In contrast, the positively charged particles did not display a consistent diffusion pattern across all sizes (Table S2). This suggests the existence of a critical particle size at which the positively charged particles begin to exhibit distinct diffusion patterns in various mucus environments. Particles smaller or larger than this threshold may fail to show a distinct pattern, mostly due to reduced diffusion efficiency through the negatively charged mucus matrix.

Confined or hindered diffusion was clearly observed for the positively charged 100 nm and 1000 nm particles, as indicated by various features, including alpha values (Fig. 4) and fractal dimension (Fig. S2). These observations align with previous studies reporting that the positively charged particles generally diffuse less efficiently in mucus than their negatively charged counterparts [36, 37]. These particles show random changes in movement due to transient binding and interactions with the complex environment [38]. Our findings reinforce previously reported phenomena, demonstrating that positively charged particles typically show a lower anomalous diffusion coefficient, reflecting reduced diffusion due to binding interactions [37, 39]. The complexity of these interactions, which deviate significantly from simple Brownian motion, makes it particularly challenging to accurately predict particle trajectories and classify their diffusion modes [36].

### Microrheology vs. Macrorheology: bulk measurements differ from size-dependent particle-scale behavior

Overall, experimental rheology reflects bulk material properties; the values presented in Fig. 6 indicate a stronger elastic and viscous behavior than trajectory-based estimates. The moduli and viscosity drop significantly when calculated from particle motion, suggesting that bulk material measurements do not directly translate to microscale particle performance [40]. This is because particle motion is influenced by microscale heterogeneities [41] (e.g., mesh size and local viscoelastic properties), which are averaged out in bulk measurements. The lower creep compliance observed in experiments implies that particles experience less resistance at the micro/nano-scale.

The particle-size-dependent analysis suggests that small particles (100 and 200 nm) experience a broad range of elastic and viscous environments. Because these particle sizes are much smaller than the characteristic length of the medium (pore size ≈ 1000 nm), they are likely to interact with other components within the pores, such as small peptides and lipids. In contrast, larger particles (1000 nm) perceive the materials within the pores as softer and more compliant. However, their movement within the mucus hydrogel is primarily restricted by the components that constitute the pore structure.

Finally, translating bulk rheological differences to the microscale particle behavior is not straightforward. Although PACM-HEC showcases different macroscopic rheological properties from PNCM and PACM-PAA, particle-scale measurements reveal overlapping and broad distributions across all mucus models. These overlapping broader distributions highlight the inherent heterogeneity within each mucus matrix, where particles experience a wide range of local environments. The particle’s interactions with the mucus matrix directly influence its micro-rheological properties. Confined or subdiffusive behavior (Fig. 4 and Fig. S2) affects particle movement, subsequently impacting trajectory analysis and the derived micro-rheological parameters. Moreover, the size and shape of the distributions indicate that the differences between PNCM and PACM-HEC, or PNCM and PACM-PAA, are less pronounced at the microscale than in bulk rheological measurements. These findings underscore the importance of capturing local heterogeneity, which bulk rheology alone cannot fully represent.

### PACM-HEC mimics native mucus diffusion for particles with a specific size and charge

In addition to enabling the identification of the material in which particles were diffusing, the diffusional fingerprinting approach proved highly valuable for characterizing overall particle-level diffusion behavior within the medium. The distribution of different features across various mucus types in this study captured distinct aspects of diffusion patterns. Specifically, the analyzed features reflected the impact of material heterogeneity and microrheology on diffusion behavior. Furthermore, categorizing datasets based on particle charge and size provided insights into how these characteristics influence diffusion patterns. Therefore, this fingerprinting approach also serves as a suitable method for identifying which artificial mucus model best mimics the diffusion pattern behavior of particles within native mucus at the microscale. A pairwise comparison of different diffusion mucus types across all features enables the extraction of such information.

In this study, we employed four different metrics for pairwise comparisons. Two of these metrics, presented in Fig. 7, revealed that for datasets involving negatively charged particles and for two involving positively charged particles (200 and 1000 nm), PACM-HEC more closely resembled native mucus in terms of microscale particle diffusion behavior. Notably, all of these datasets—except for the 1000 nm positively charged particle case—also exhibited higher classification accuracy (77–86%) in identifying the diffusion matrix.

We also identified viscosity and Gaussianity as two of the most influential features driving the similarity between PNCM and PACM-HEC compared with the similarity between PNCM and PACM-PAA. Previous studies have shown that macroscopic viscosity and mesh size do not differ significantly among PNCM, PACM-HEC, and PACM-PAA [19]. However, PACM-PAA exhibits a significantly higher negative zeta potential (−45 to −55 mV) than PNCM (−15 to −22 mV) and PACM-HEC (−19 to −23 mV) (Fig. 1). Although these differences in surface charge do not markedly influence bulk rheological measurements, they do affect particle-level dynamics. As a result, particle motion in PACM-HEC more closely resembles that in PNCM.

Electrostatic interactions may exert a stronger influence on the diffusion patterns for positively charged particles, particularly those below (100 nm) or above (1000 nm) a certain critical size threshold. This effect is also reflected in the relatively low classification accuracies observed for these cases (67% and 57%, respectively). While PACM-PAA appears to more closely resemble PNCM for the 100 nm positively charged particles, it may not fully capture the mechanisms governing their effective diffusion behavior. Therefore, with the considerations of physicochemical properties (Fig. 1b) in particular the zeta potential, together with the result from class similarity (Fig. 7), PACM-HEC is the best candidate to mimic PNCM compared to PACM-PAA.

### Study limitations and future perspective

This study, despite its extensive scope, has several limitations. We utilized polystyrene nano- and microparticles as models for undissolved drug particles; future studies should incorporate clinically relevant drug delivery particles to better capture real-world behaviors. Another limitation lies in the artificial colonic mucus model itself, which has a simplified composition of proteins and lipids compared to native mucus. This simplification might affect mucus physicochemical properties (such as the charge-shielding effects), which consequently influence particle diffusion. Additionally, the artificial mucus used here is based primarily on porcine mucus, a preclinical species of increased interest in drug absorption studies. Future research should therefore explore how more complex protein and lipid compositions impact particle diffusion, as well as perform comparative analyses between human and porcine mucus for translational purposes.

## Conclusion

This study demonstrated that diffusional fingerprinting is a viable technique for characterizing both particle-level diffusion behavior and the rheological properties of the diffusion medium using particle-level trajectory data. It successfully differentiated the diffusion patterns across particles of various sizes and charges, while identifying the corresponding diffusion medium. This approach not only identified which particles diffuse most efficiently in native mucus but also determined which artificial mucus model best mimics the native environment for those particles. Incorporation of microrheological properties to improve diffusional fingerprinting techniques is also recommended, while evaluation of biosimilar mucus model would also benefit from this technique. Selecting the most suitable artificial mucus model will improve the accuracy of drug diffusion studies, thereby facilitating the development of more effective drug delivery systems and optimizing therapeutic outcomes, particularly for advanced drug delivery systems targeting absorption throughout the gastrointestinal tract.

## Supplementary Information

Supplementary Material 1

## Data Availability

Data is available upon request.
