# Supplementary material for: Machine learning framework for investigating nano- and micro-scale particle diffusion in colonic mucus
Source: J Nanobiotechnology. 2025 Aug 22;23:583. doi: 10.1186/s12951-025-03659-6 (PMC12372352; doi:10.1186/s12951-025-03659-6)
Supplement: Supplementary file 1 — Supplementary Material 1 [file 12951_2025_3659_MOESM1_ESM.docx]

Machine learning framework for investigating nanoparticle diffusion in colonic mucus

# **Authors**

Marco Tjakra^a,b^, Kristína Lidayová^c,d^, Christophe Avenel^c,d^, Christel AS Bergström^a,b^, and Shakhawath Hossain^a,b*^

^a^Department of Pharmacy, Uppsala Biomedical Center, Uppsala University, 751 23 Uppsala, Sweden

^b^The Swedish Drug Delivery Center, Department of Pharmacy, Uppsala University, Box 580, SE-751 6 23 Uppsala, Sweden

^c^Department of Information Technology, Uppsala University, Uppsala, Sweden

^d^BioImage Informatics Facility, Science for Life Laboratory, SciLifeLab, Sweden

*Corresponding author:

E-mail: [shakhawath.hossain@uu.se](mailto:shakhawath.hossain@uu.se)
Visiting address: Biomedicinskt Centrum BMC, Husargatan 3
Postal address: Box 580, 751 23 Uppsala

Contents

Method S1 Image acquisition, preprocessing, and particle tracking with TrackPy 3

Figure S1 F1-scores for each classifier and data category 6

Figure S2 Distribution fractal dimensions 7

Figure S3 Distribution of 20 features output for the ‘All Data’ 8

Figure S4 Distribution of 20 features output for the ‘Pos. Charged’ 9

Figure S5 Distribution of 20 features output for the ‘Neg. Charged’ 10

Figure S6 Distribution of 20 features output for the ‘Pos. Charged’ (100nm) 11

Figure S7 Distribution of 20 features output for the ‘Neg. Charged’ (100nm) 12

Figure S8 Distribution of 20 features output for the ‘Pos. Charged’ (200nm) 13

Figure S9 Distribution of 20 features output for the ‘Neg. Charged’ (200nm) 14

Figure S10 Distribution of 20 features output for the ‘Pos. Charged’ (1000nm) 15

Figure S11 Distribution of 20 features output for the ‘Neg. Charged’ (1000nm) 16

Figure S12 Top five features with the largest positive differences 17

Figure S13 Comparison of similarity metrics (area overlap and cosine similarity) 18

Figure S14 Comparison of similarity metrics (KL divergence and Euclidean distance) 19

Figure S15 Data analysis workflow incorporating machine learning 20

Table S1 Video microscopy of mucus groups and replicates 21

Table S2 Hydrodynamic sizes and zeta potential of polystyrene nanoparticles 22

Table S3 F1-accuracy comparison using histogram-based gradient boosting for various datasets 23

# Supplementary Methods

**Method S1: Image acquisition, preprocessing, and particle tracking with TrackPy**

The algorithm begins by identifying potential nanoparticles by detecting local maxima in the filtered images. Real particles are distinguished from spurious ones by applying a brightness threshold (“min_mass” parameter). TrackPy then links particles frame-to-frame using a predictive nearest-neighbor search algorithm. This is coupled with a cost-minimizing assignment approach to improve tracking accuracy. Trajectories shorter than the number of frames specified in the “trajectory duration” parameter are considered spurious and removed from analysis. TrackPy further refines particle localization to subpixel precision using a Gaussian mask fitting method. The resulting nanoparticle positions and trajectories are stored in pandas DataFrames for downstream analysis. Trajectory analysis includes the calculation of MSD. These MSD profiles are then fitted to a power law using TrackPy’s “fit_powerlaw” function, which performs linear regression in logarithmic space.

For loading video frames into Python, the Python Image Sequence package was employed to efficiently handle image sequences.

To ensure optimum tracking using TrackPy, the following parameters and their corresponding values were applied:

| **Parameter** | **Value** | **Explanation** |
| --- | --- | --- |
| dot_size | 11 | Approximate diameter (in pixels) of the particle to be detected. This parameter controls the size of features to be identified. |
| min_mass | 500 | Minimum integrated brightness (that is, the sum of pixel intensities) required for a feature to be recognized as a particle. The default value is typically 100 for integer images and 1 for float images, but higher thresholds help filter out noise. |
| displacement  trajectory_duration | 5  10 | Maximum distance (in pixels) that a particle can travel between consecutive frames during linking.  Minimum duration (in number of frames) that a trajectory must persist to be retained for analysis. Trajectories shorter than, for example, 10 frames are considered spurious and excluded. |
| frame_memory | 10 | Maximum number of consecutive frames during which a particle can disappear and still be considered the same particle upon reappearance. |
| microns_per_pixel | 0.114 | Conversion factor from image pixels to physical units (microns). This value is used for scaling trajectories and compute MSD calculations. |
| frames_per_second | 1 | Frame rate of the video, expressed in frames per second. This parameter is essential for accurate time scaling in dynamic analyses like MSD. |

After nanoparticle detection was completed, real particles were distinguished from spurious ones using an intensity-based threshold. The total brightness (“mass”) served as the primary thresholding parameter. Particle features were extracted across 100 frames of each video. The “signal” strength and uncertainty estimates were derived based on previous research [23]. Trajectories of each detected particle were recorded. Ephemeral trajectories—those lasting only a few frames—are typically spurious and excluded from analysis. The “filter_stubs” function was applied to retain only those trajectories that persisted for a specified number of frames. DataFrame reports were generated and stored for further analysis. Trajectory analysis included the calculation of MSD. The ensemble-averaged MSD was then fitted to a power law using the “fit_powerlaw” function, which performs a linear regression in logarithmic space.

# Supplementary Data


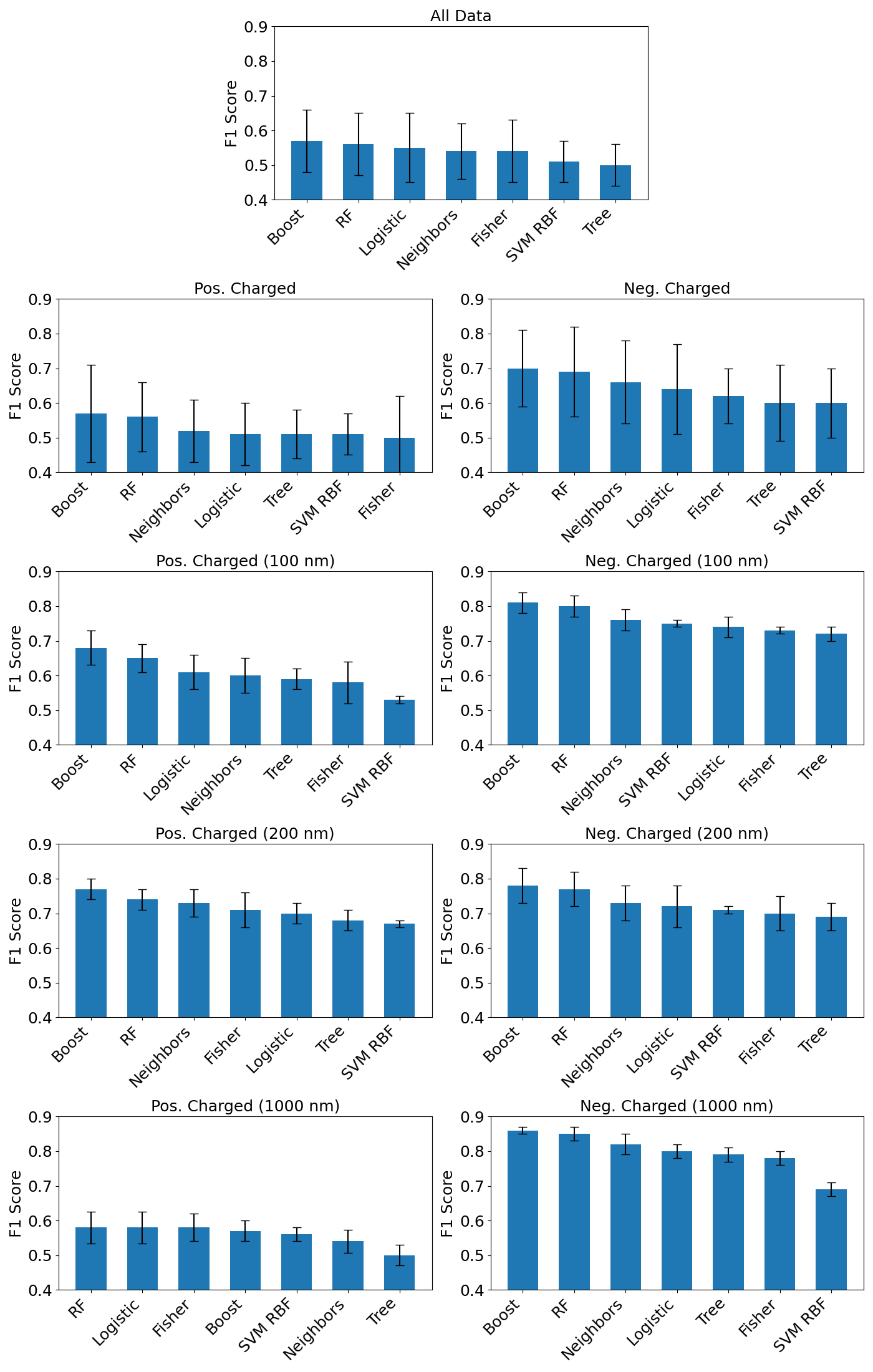
Supplementary Figure S1: F1-scores for each classifier and data category

Supplementary Figure S2: The distribution of fractal dimensions (one from twenty different features) for six data sets consisting of particles with specific sizes and charges


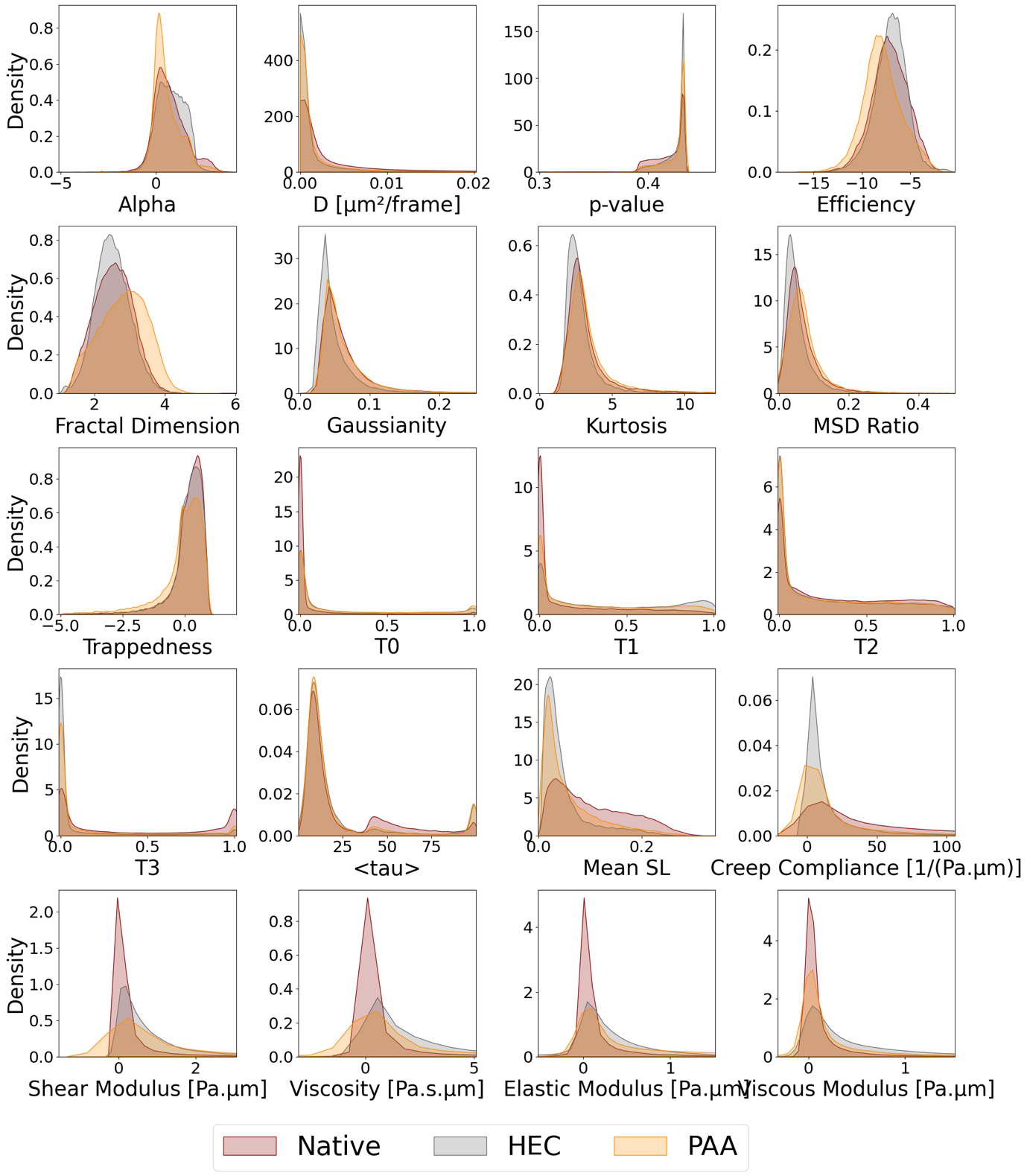


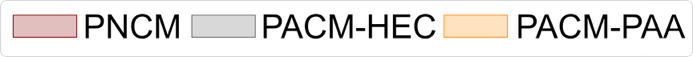


Supplementary Figure S3: Distribution of 20 features output by the diffusional fingerprinting for three different mucus models for the ‘All Data’ dataset. The density distributions in the figure were computed using kernel density estimation (KDE) with the Seaborn kdeplot function.


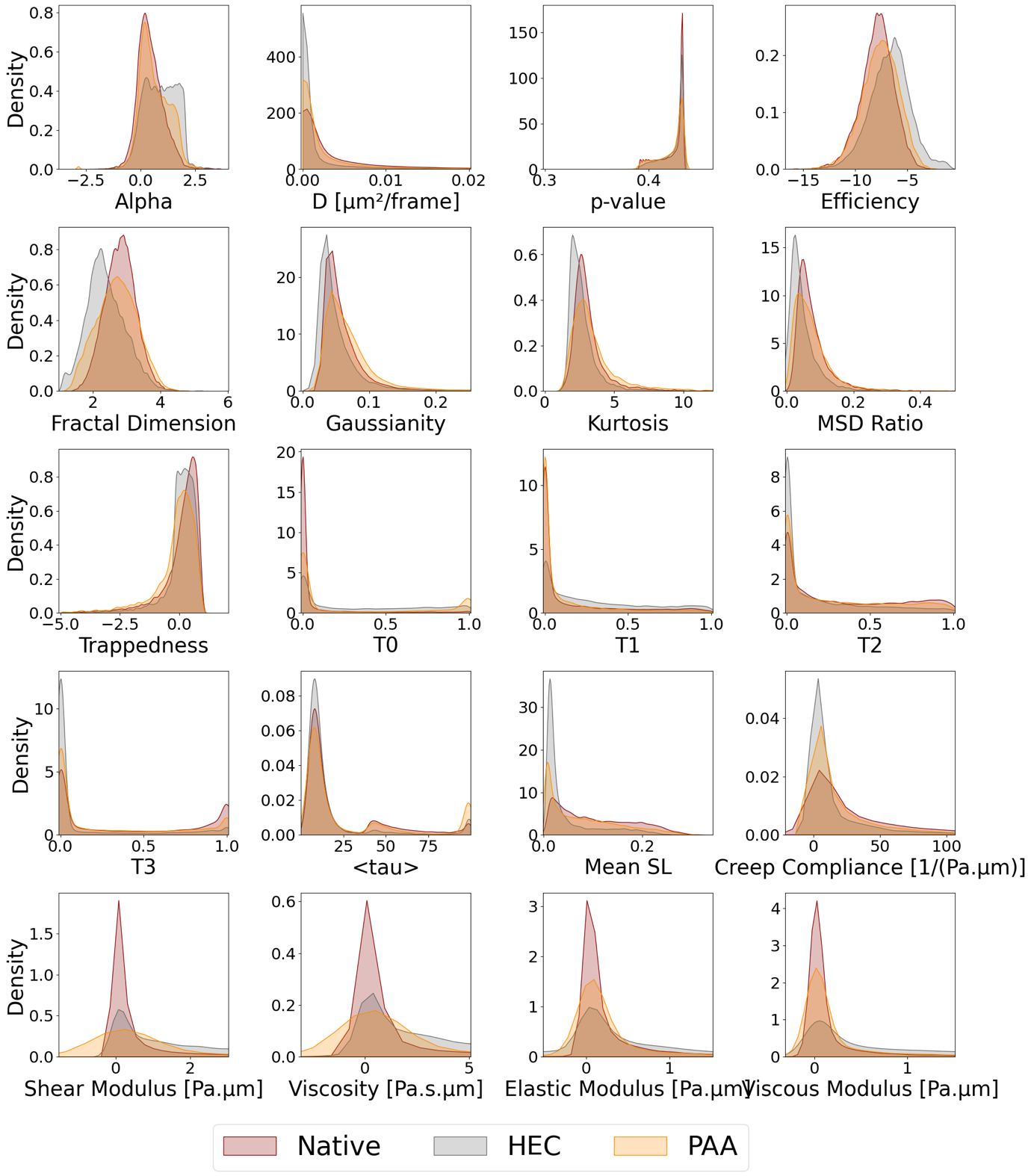


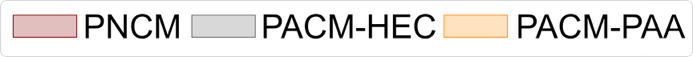


Supplementary Figure S4: Distribution of 20 features output by the diffusional fingerprinting for three different mucus models for the ‘Positively charged’ data category.


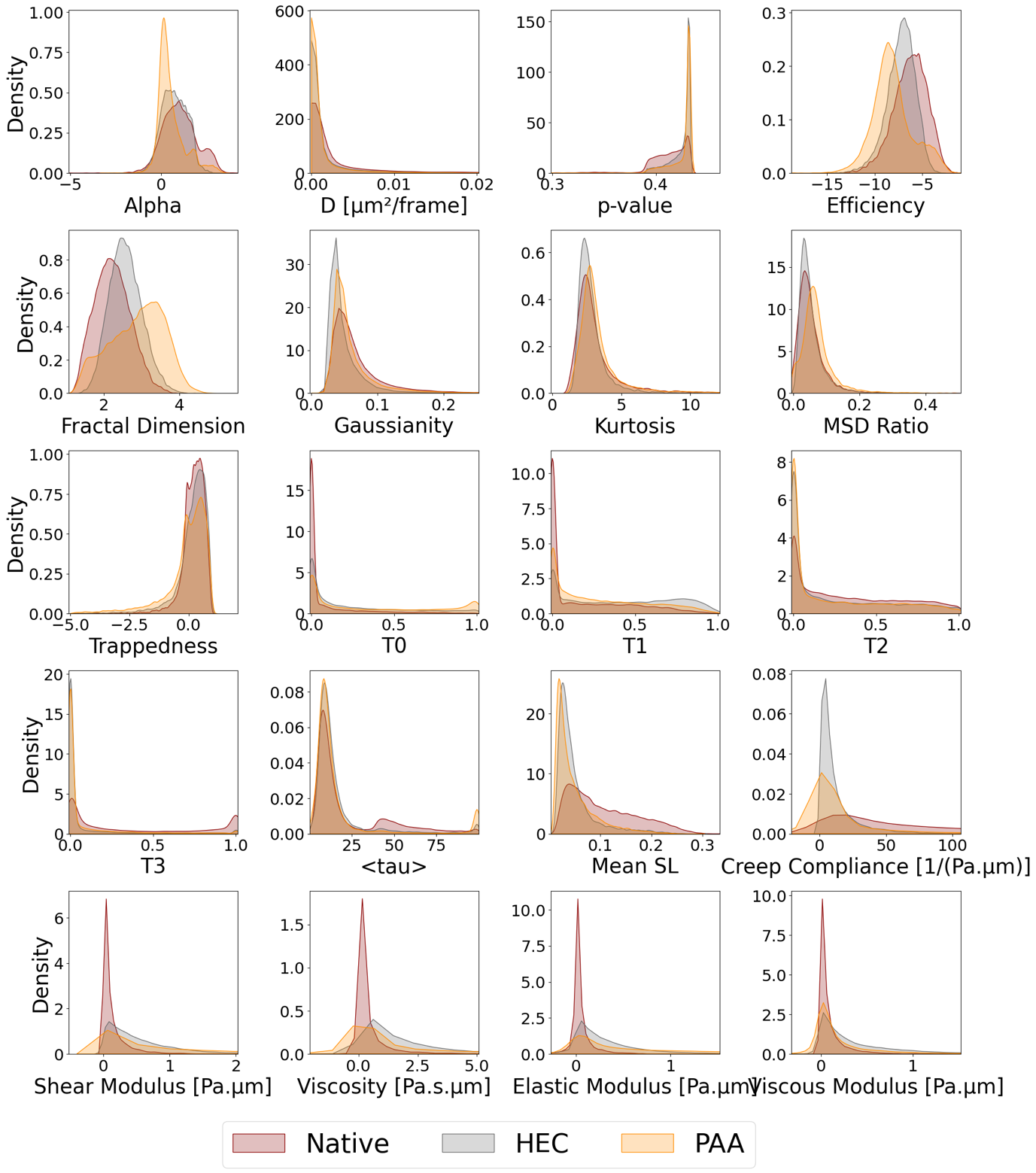


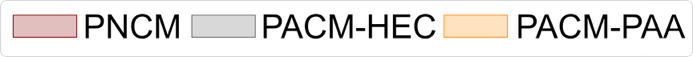


Supplementary Figure S5: Distribution of 20 features output by the diffusional fingerprinting for three different mucus models for the ‘Negatively charged’ data category.


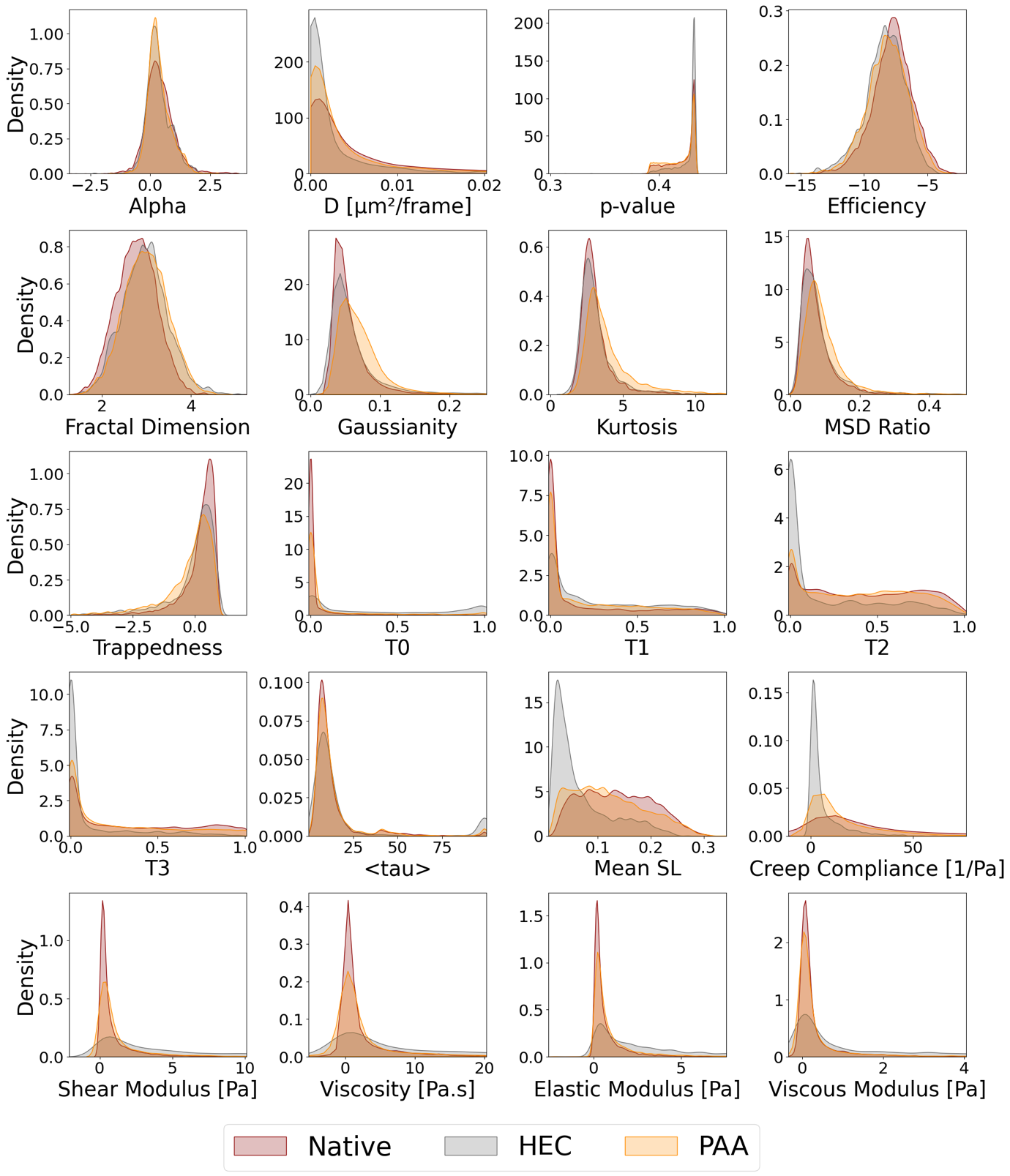


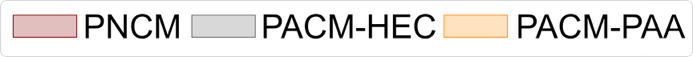


Supplementary Figure S6: Distribution of 20 features output by the diffusional fingerprinting for three different mucus models for the ‘Positively charged 100 nm’ data category.


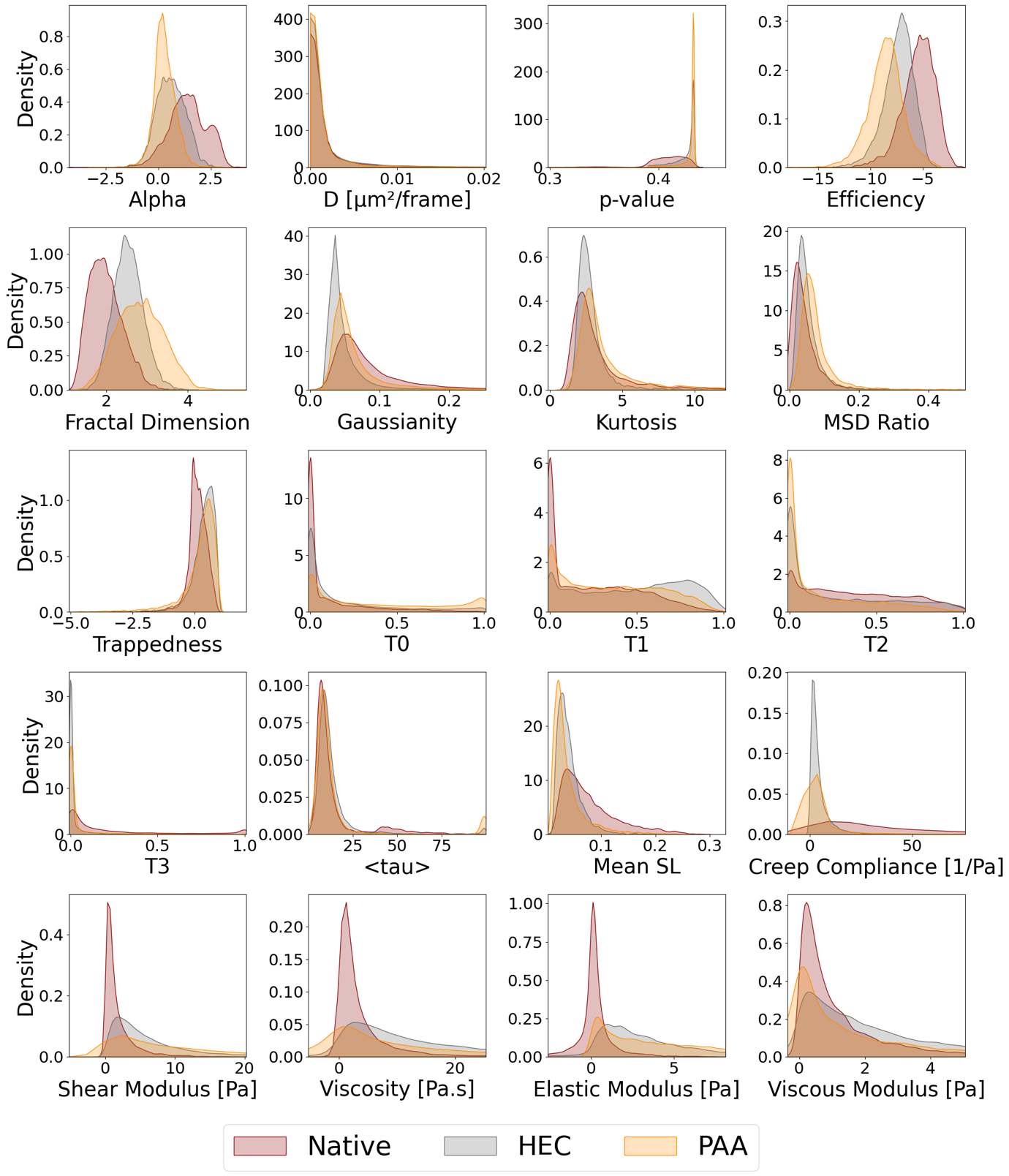


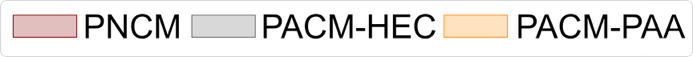


Supplementary Figure S7: Distribution of 20 features output by the diffusional fingerprinting for three different mucus models for the ‘Negatively charged 100 nm’ data category.


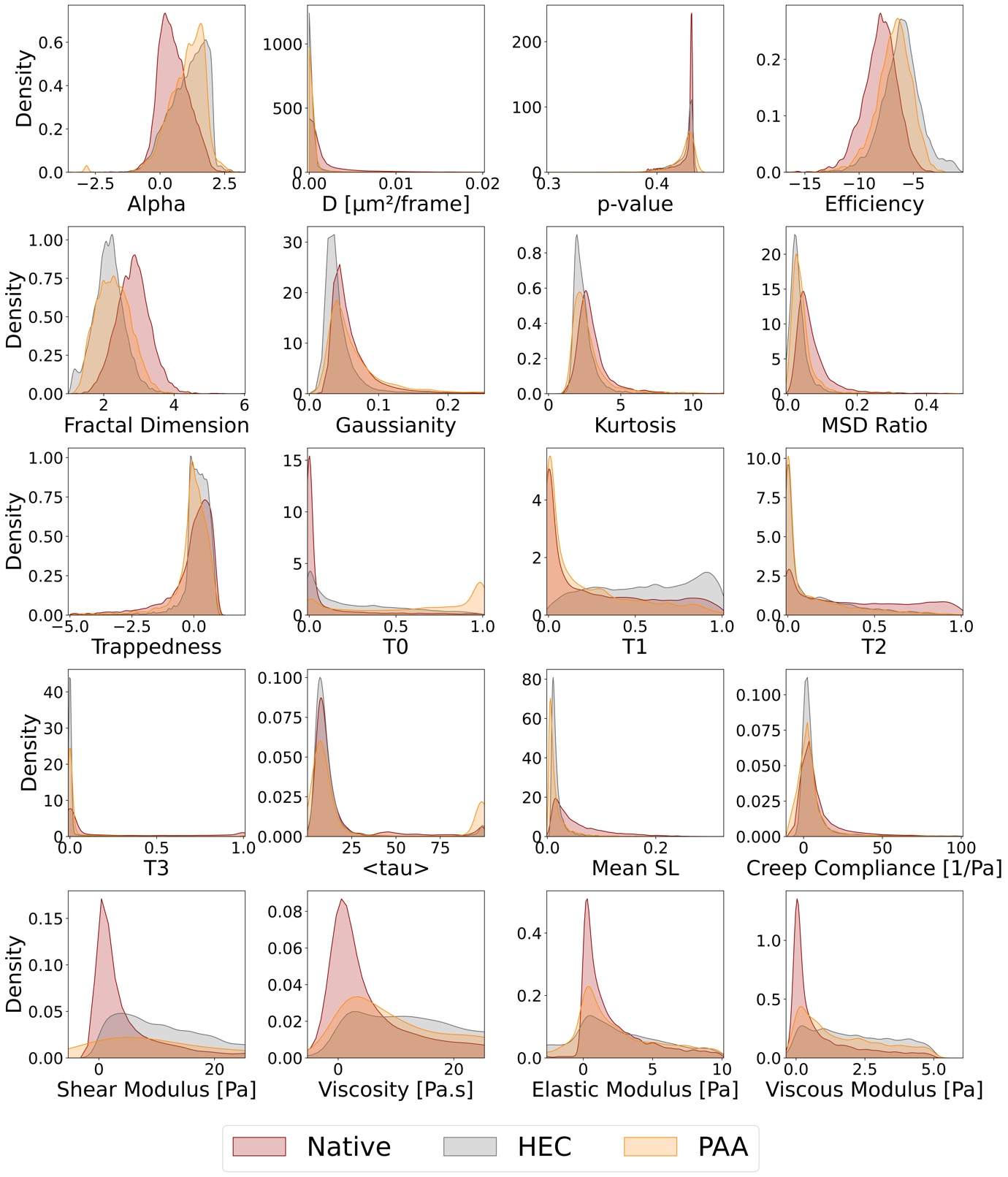


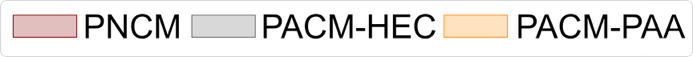


Supplementary Figure S8: Distribution of 20 features output by the diffusional fingerprinting for three different mucus models for the ‘Positively charged 200 nm’ data category.


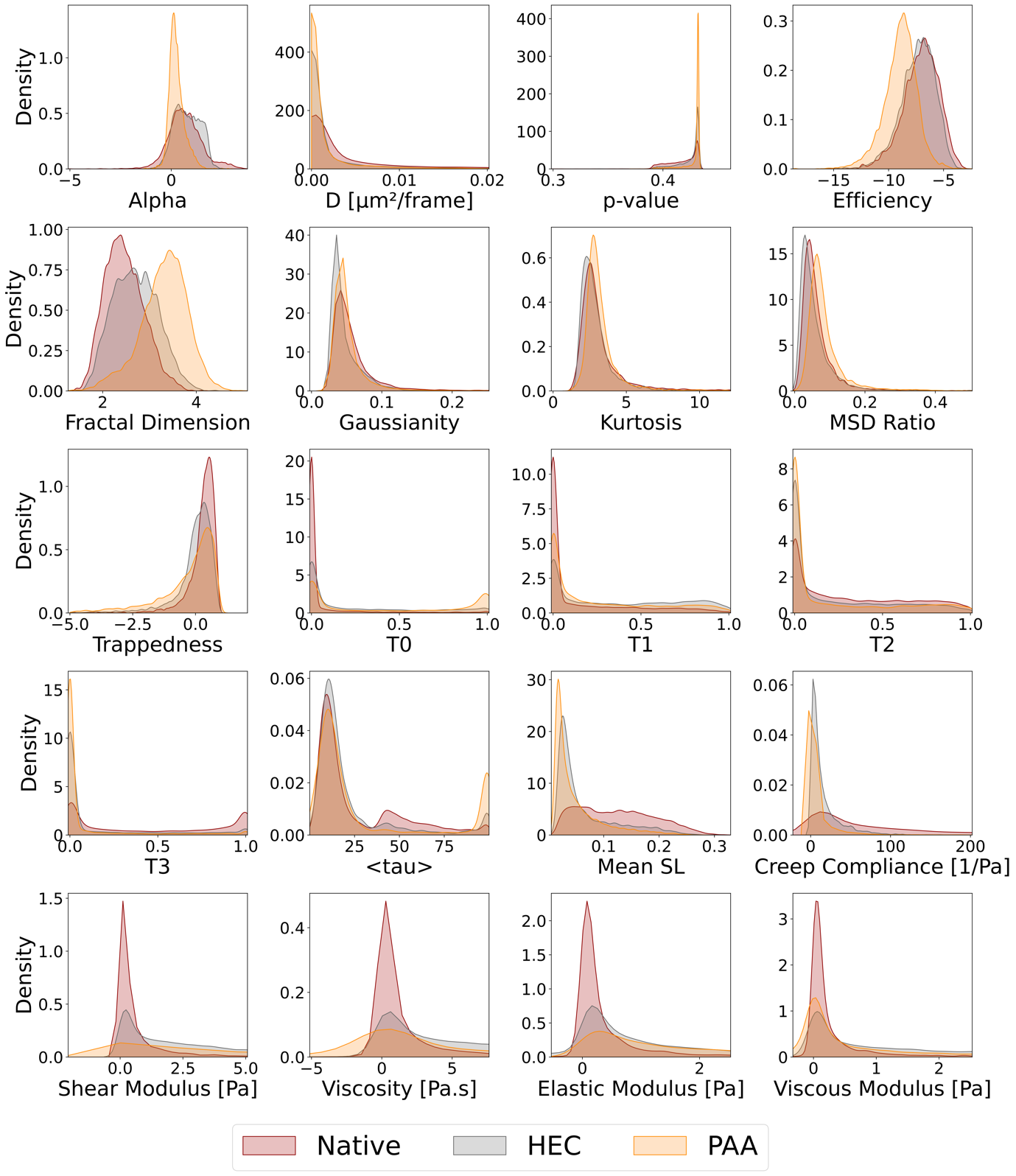


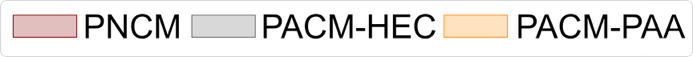


Supplementary Figure S9: Distribution of 20 features output by the diffusional fingerprinting for three different mucus models for the ‘Negatively charged 200 nm’ data category.

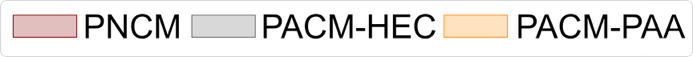


Supplementary Figure S10: Distribution of 20 features output by the diffusional fingerprinting for three different mucus models for the ‘Positively charged 1000 nm’ data category.

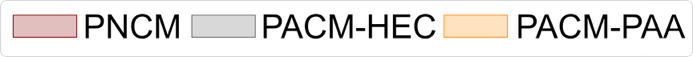


Supplementary Figure S11: Distribution of 20 features output by the diffusional fingerprinting for three different mucus models for the ‘Negatively charged 1000 nm’ data category.

Supplementary Figure S12: The top five features with the largest positive differences in area overlap similarity values for each dataset, grouped by particle size and surface charge.

The difference was calculated as:

Difference = Area overlap similarity (PNCM–PACM-HEC) − Area overlap similarity (PNCM–PACM-PAA).

Positive values indicate features that are more similar between PNCM and PACM-HEC than between PNCM and PACM-PAA.


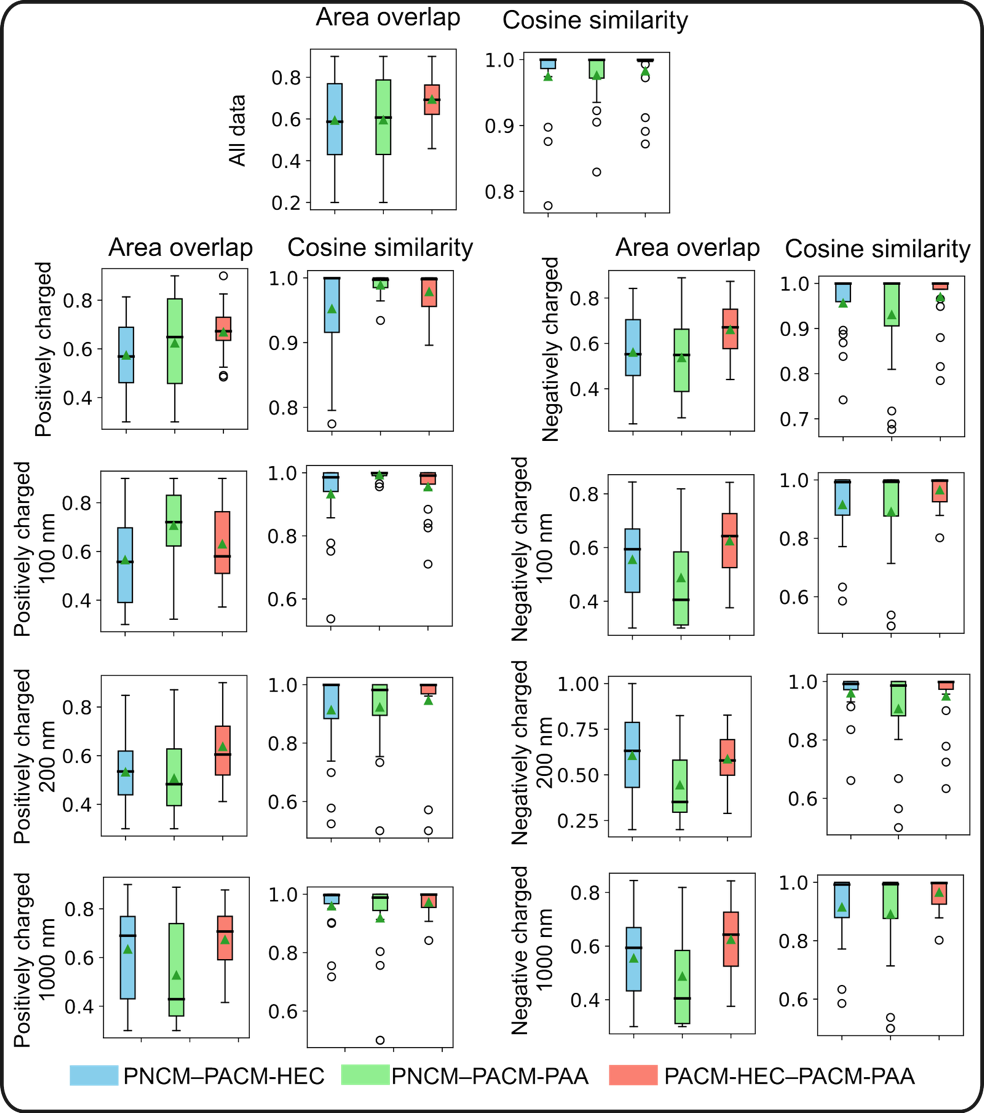


Supplementary Figure S13: Comparison of similarity metrics for pairwise comparisons among the three mucus models—PNCM, PACM-HEC, and PACM-PAA—using area overlap and cosine similarity across all datasets. The analysis considers all computed features. Each box in the plot represents the interquartile range, with the black horizontal line indicating the median similarity value. Green triangles represent the mean similarity values, providing insights into the overall central tendency. Black circles outside the whiskers denote outliers, signifying values that fall markedly outside the main distribution. Color coding corresponds to the three pairwise comparisons: PNCM–PACM-HEC (sky blue), PNCM–PACM-PAA (light green), and PACM-HEC–PACM-PAA (salmon).


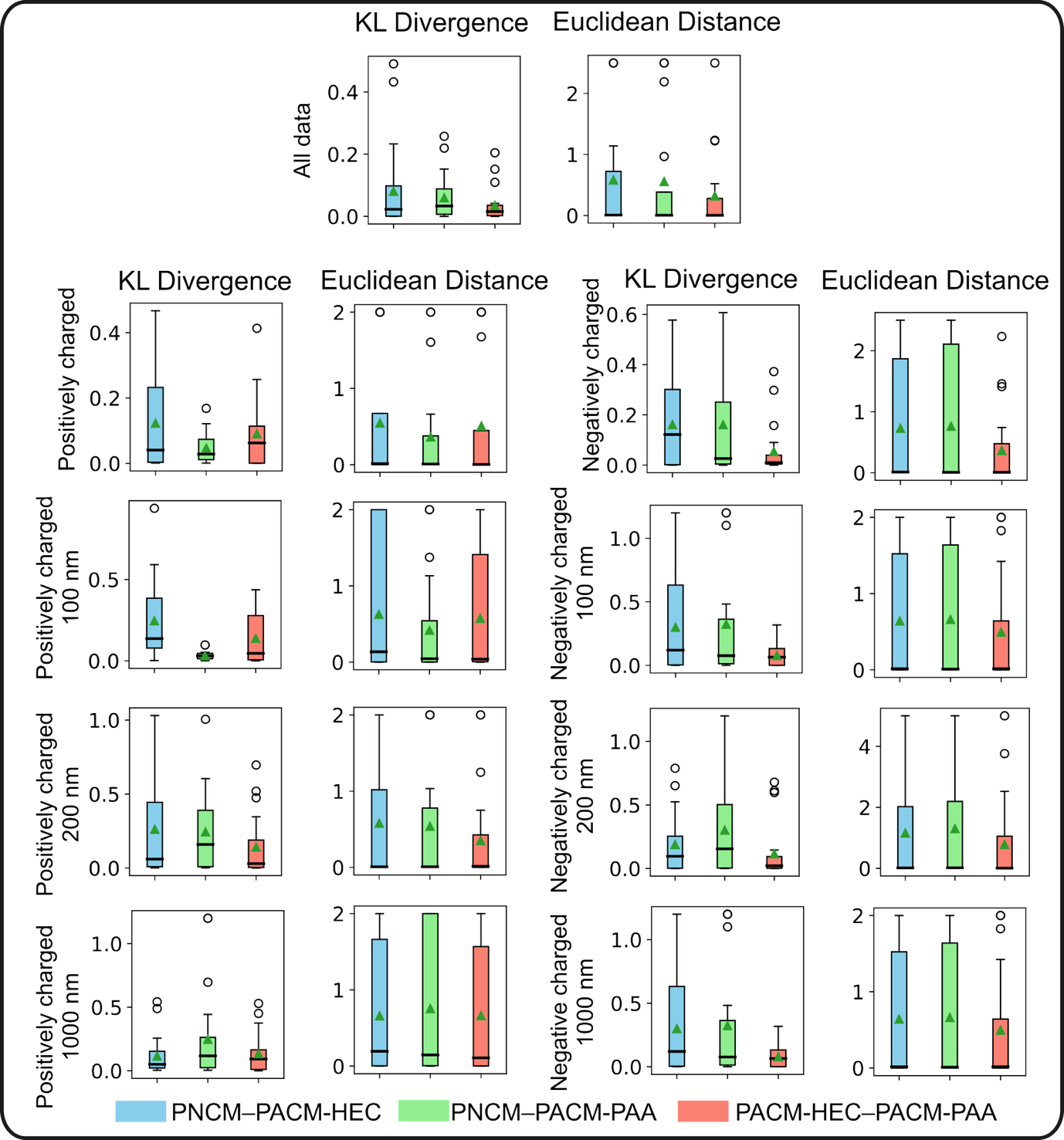


Supplementary Figure S14: Comparison of similarity metrics for the pairwise comparisons of the three mucus models—PNCM, PACM-HEC, and PACM-PAA—using KL Divergence and Euclidean Distance across all datasets. The analysis considers all computed features. Each box in the plot represents the interquartile range (IQR), with the black horizontal line inside the box indicating the median similarity value. The green triangles represent the mean similarity values, providing insight into the overall central tendency. The black circles outside the whiskers denote outliers, representing values that fall significantly outside the main distribution. The color coding corresponds to the three pairwise comparisons: PNCM–PACM-HEC (sky blue), PNCM–PACM-PAA (light green), and PACM-HEC–PACM-PAA (salmon).

Supplementary Figure S15: Data analysis workflow incorporating machine learning. Started by characterizing particles trajectories data based on features (Fig. S2-S10), followed by application of machine learning models training and validation to generate models. Highest accuracy is GradBoost algorithm, from which analysis of SHAP and KDE were performed to classify important features. From the MSD-fitting, α-value which indicates particles diffusion mode were also analyzed.

#### Table S1. Video microscopy of mucus groups and replicates

|  | Type and particle size (nm) | Number of videos (replicates) | Number of trajectories with more than 40 frames |
| --- | --- | --- | --- |
| PNCM | Amine (Pos. Charge) |  |  |
|  | 100 | 10 | 6353 |
|  | 200 | 10 | 7076 |
|  | 1000 | 5 | 1627 |
|  | Carboxylate (Neg. Charge) |  |  |
|  | 100 | 10 | 6507 |
|  | 200 | 10 | 6343 |
|  | 1000 | 5 | 784 |
| PACM-HEC | Amine (Pos. Charge) |  |  |
|  | 100 | 7 | 1330 |
|  | 200 | 8 | 5788 |
|  | 1000 | 6 | 1573 |
|  | Carboxylate (Neg. Charge) |  |  |
|  | 100 | 7 | 8904 |
|  | 200 | 9 | 5907 |
|  | 1000 | 5 | 1556 |
| PACM-PAA | Amine (Pos. Charge) |  |  |
|  | 100 | 7 | 5572 |
|  | 200 | 9 | 4163 |
|  | 1000 | 5 | 810 |
|  | Carboxylate (Neg. Charge) |  |  |
|  | 100 | 6 | 6760 |
|  | 200 | 7 | 7219 |
|  | 1000 | 5 | 2967 |

#### Table S2. Hydrodynamic sizes and zeta potential of polystyrene nanoparticles in the particle tracking experiments. Measurements were performed in triplicates.

| Type and particle size (nm) | Zeta-potential (mV) | SD (±) | Hydrodynamic sizes (nm) | SD (±) |
| --- | --- | --- | --- | --- |
| Amine |  |  |  |  |
| 100 | 58.8 | 1.8 | 95.9 | 3.8 |
| 200 | 22.3 | 2.2 | 283.9 | 19.2 |
| 1000 | -3.6 | 0.4 | 1315.9 | 467.7 |
| Carboxylate |  |  |  |  |
| 100 | -43.7 | 5.2 | 111.8 | 2.6 |
| 200 | -51.8 | 2.5 | 237.5 | 13.5 |
| 1000 | -37.7 | 1.9 | 2440.8 | 1818.5 |

#### Table S3. F1-accuracy comparison using histogram-based gradient boosting for various datasets. Accuracy values are reported for models using all features (including HMM-based and rheological), excluding only rheological features (1), and excluding both HMM-based and rheological features (2).

| Data categorization | Accuracy (%) | | |
| --- | --- | --- | --- |
|  | With all features (current study) | Without rheological features | Without HMM-based and rheological features |
| All Data | 57 | 57 | 57 |
| Positively charged | 57 | 56 | 57 |
| Negatively charged | 70 | 69 | 69 |
| Positively charged 100 nm | 67 | 61 | 59 |
| Negatively charged 100 nm | 81 | 81 | 80 |
| Positively charged 200 nm | 77 | 75 | 74 |
| Negatively charged 200 nm | 78 | 76 | 76 |
| Positively charged 1000 nm | 58 | 57 | 57 |
| Negatively charged 1000 nm | 86 | 85 | 80 |
